# The Majority of Typhoid Toxin-Positive *Salmonella* Serovars Encode ArtB, an Alternate Binding Subunit

**DOI:** 10.1128/mSphere.01255-20

**Published:** 2021-01-06

**Authors:** A. Gaballa, R. A. Cheng, A. S. Harrand, A. R. Cohn, M. Wiedmann

**Affiliations:** a Department of Food Science, Cornell University, Ithaca, New York, USA; b Department of Microbiology, Cornell University, Ithaca, New York, USA; University of Kentucky

**Keywords:** typhoid toxin, nontyphoidal *Salmonella*, cytolethal distending toxin, ArtB

## Abstract

Salmonella enterica encodes a wide array of virulence factors. One novel virulence factor, an A_2_B_5_ toxin known as the typhoid toxin (TT), was recently identified among a variety of S. enterica serovars. While past studies have shown that some serovars encode both the TT (active subunits CdtB and PltA and binding subunit PltB) and a second binding subunit (ArtB), these serovars were thought to be the exception. Here, we show that genes encoding the TT are detected in more than 100 serovars representing distinct phylogenetic lineages of S. enterica subsp. *enterica*, although clade B and section Typhi are significantly more likely to encode TT genes than serovars from other clades. Furthermore, we show that 81% of these TT-positive serovars also encode *artB*, suggesting that the cooccurrence of both toxin binding subunits is considerably more common than previously thought. A combination of *in silico* modeling, bacterial two-hybrid system screening, and tandem affinity purification (TAP) of toxin subunits suggests that ArtB and PltB interact *in vitro*, at least under some growth conditions. While different growth conditions yielded slightly higher transcript abundances of *artB* and *pltB*, both genes had their highest relative transcript abundances when *Salmonella* was grown under low-Mg^2+^ conditions, suggesting that ArtB and PltB may compete for inclusion in the TT. Together, our results suggest that ArtB likely plays an important and previously underappreciated role in the biology of the TT produced by typhoidal and nontyphoidal *Salmonella*.

**IMPORTANCE** While previous reports had suggested that the typhoid toxin (TT) could potentially use ArtB as an alternate binding subunit, this was thought to play a minor role in the evolution and biology of the toxin. In this study, we establish that both TT genes and *artB* are widespread among Salmonella enterica subsp. *enterica*, suggesting that TT likely plays a broader role in *Salmonella* virulence that extends beyond its proposed role in typhoid fever. Furthermore, our data suggest the selective maintenance of both toxin binding subunits, which may compete for inclusion in the holotoxin. Last, our data support the importance of characterizing diverse nontyphoidal *Salmonella* (NTS) serovars, as the presence of classically defined typhoidal virulence factors among NTS serovars continues to challenge the typhoid-nontyphoid *Salmonella* paradigm.

## Abstract (precis)

While previous reports had suggested that the typhoid toxin (TT) could potentially use ArtB as an alternate binding subunit, this was thought to play a minor role in the evolution and biology of the toxin. In this study, we establish that both TT genes and *artB* are widespread among Salmonella enterica subsp. *enterica*, suggesting that TT likely plays a broader role in *Salmonella* virulence that extends beyond its proposed role in typhoid fever.

## INTRODUCTION

Salmonella enterica is an important cause of foodborne illness worldwide, contributing an estimated 88 million (95% uncertainty interval, 35 million to 234 million) foodborne infections each year (1). Given the appreciable diversity of S. enterica with >2,600 known serovars (2), efforts to understand this pathogen have largely focused on categorizing serovars into typhoidal, paratyphoidal, and nontyphoidal types (3, 4) and studying select representatives of each (5). Arguably the two best-characterized serovars are S. enterica serovar Typhi and S. enterica serovar Typhimurium, representing typhoidal and nontyphoidal serovars, respectively. *S.* Typhi, the causative agent of typhoid fever, is associated with an invasive extraintestinal infection (4), while infection with nontyphoidal serovars usually results in self-limiting gastroenteritis (4), although infection with some serovars is associated with a significantly higher likelihood of invasive disease (6). While these two serovars have been, and continue to be, the proverbial workhorses that have greatly progressed our understanding of *Salmonella* pathogenesis, the frequency of human infections with other less-studied serovars continues to increase (7–11). Regardless, investigations into *Salmonella* pathogenesis with *S.* Typhi and *S.* Typhimurium have identified an array of virulence factors and adaptations that allow diverse *Salmonella* serovars to successfully colonize and cause disease in a range of hosts (5, 12, 13).

The typhoid toxin (TT), which was originally discovered and characterized in *S.* Typhi (14, 15), has been proposed as a key player in the development of typhoid fever, acting at both the single-cell and systemic levels in models of infection (14, 16–19). The TT incorporates the nuclease activity of the CdtB subunit of the cytolethal distending toxin (CDT) (14, 20) with the mono-ADP-ribosyltransferase activity of the pertussis toxin (called PltA), resulting in a hybrid toxin that induces a DNA damage response (DDR) in eukaryotic cells, leading to an accumulation of cells in the G_2_/M cell cycle phase (17, 18) and subsequent senescence (21, 22). The resulting damaged DNA is proposed to play a role in both disease manifestation (18) and colonization and persistence of the toxin-producing bacteria in the host (19, 23), although its role in acute typhoid fever remains uncertain (24).

The binding subunit of the TT was originally characterized as a pentameric ring of PltB monomers (18). ArtB, a homolog of PltB, was later identified in select *S.* Typhimurium DT104 isolates (25). As a proof of concept, Gao et al. showed that ArtB from *S.* Typhimurium DT104 can assemble into a homopentamer *in vitro* and further confirmed that the ArtB homopentamer can interact with CdtB and PltA from *S.* Typhi to form a biologically active holotoxin *in vitro* and *in vivo* (26). While both ArtB and PltB bind to glycan residues on sialic acids on host cells, ArtB is able to bind both *N*-acetylneuraminic acid (Neu5Ac)- and *N*-glycolylneuraminic acid (Neu5Gc)-terminated glycans on sialic acids, whereas PltB preferentially binds Neu5Ac-terminated glycans (18, 26). Gao et al. proposed that the expanded binding repertoire of ArtB could reflect the broad host range of *S.* Typhimurium DT104, despite the fact that *S.* Typhimurium isolates do not encode *cdtB*, *pltA*, or *pltB* (26).

We and others have previously established that multiple nontyphoidal serovars encode both *artB* and *pltB* (16, 27, 28), including S. enterica serovar Javiana, the 4th most commonly isolated serovar from human clinical cases of salmonellosis in the United States (7). Here, we show that the majority of TT-encoding serovars also encode *artB* and that PltB and ArtB may compete for inclusion in the binding subunit of the holotoxin.

## RESULTS

### TT genes are significantly overrepresented among specific clades of S. enterica.

**FIG 1:**
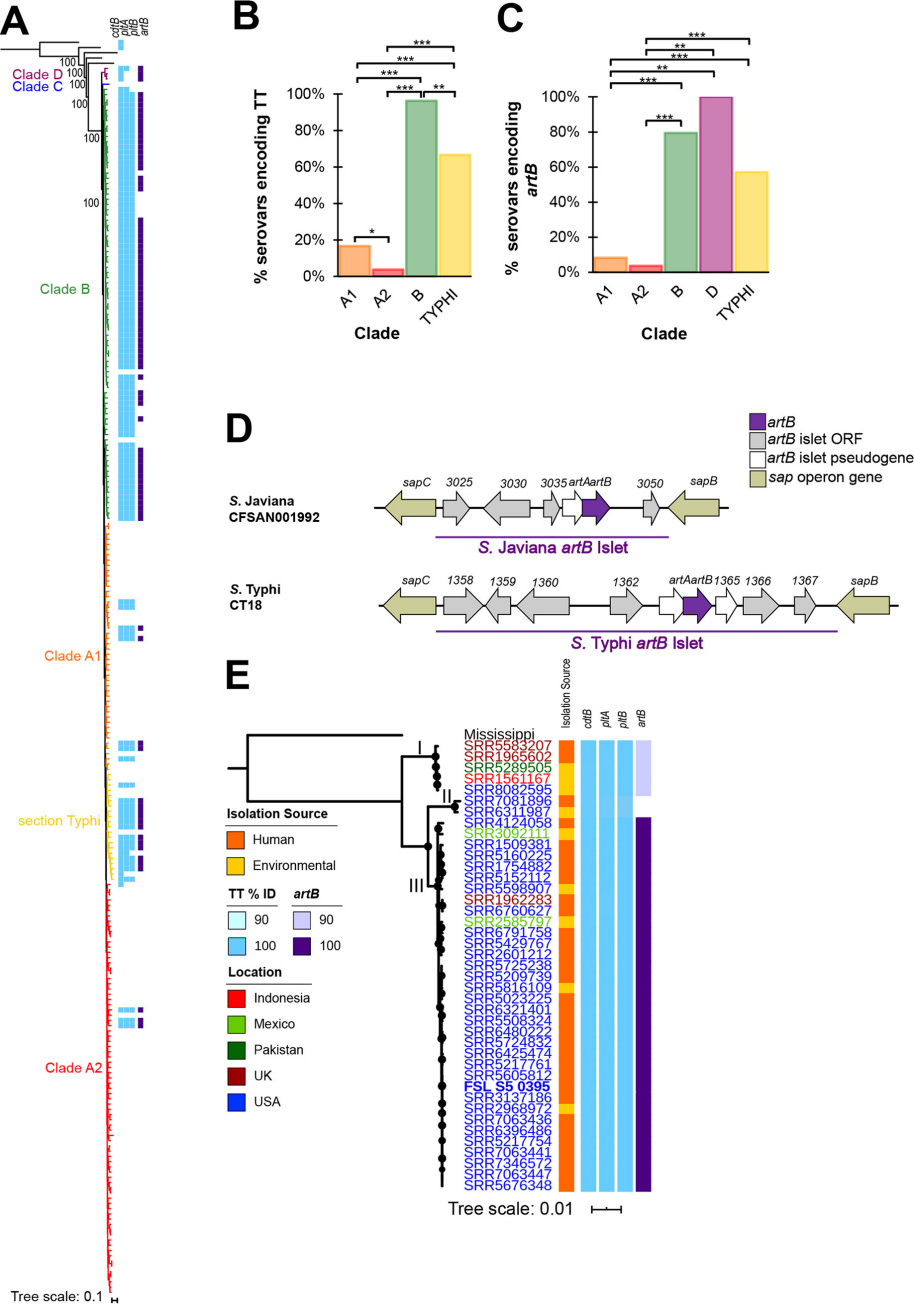
Serovars in some phylogenetic clades are significantly more likely to encode TT genes and *artB*. (A) Reconstruction of phylogeny of 235 S. enterica subsp. *enterica* serovars and five additional subspecies based on the comparison of 13,181 core SNPs. Maximum likelihood analysis was computed using a general time-reversible model with gamma distribution in RAxML (66). Bootstrap values for branchpoints between subspecies are shown (based on 100 bootstrap repetitions). Branches are colored to represent the clade of the isolate as reported previously (29) and the detection of TT genes (*cdtB*, *pltA*, and *pltB*; shown in light blue) and *artB* (shown in purple) among S. enterica isolates using 85% nucleotide identity and 90% query coverage as criteria for detection. S. enterica subsp. *arizonae* was used to root the phylogeny. Histograms summarizing the proportion of serovars in each clade for which all TT genes (*cdtB*, *pltA*, and *pltB*) (B) or *artB* (C) was detected with blastn (i.e., the additional 6 S. enterica subsp. *enterica* serovars having putative *pltA* and *pltB* were not included in this analysis); note that clade C is not shown because TT genes and *artB* were not detected in these serovars. *, *P* < 0.05; **, *P* < 0.001; ***, *P* <0.0001, FDR corrected for pairwise comparisons of Fisher’s exact tests. (D) Comparison of the *artB*-islet in *S.* Javiana CFSAN001992 and *S.* Typhi CT18; ORFs and genes are named according to annotations from NCBI. The *artB* islet is denoted by the purple horizontal bar. ORFs that are at least 100 nucleotides (nt) in length are shown in gray (those <100-nt long were classified as pseudogenes and are shown in white), while genes shown in gold represent *sap* operon genes that flank the *artB* islet; *artB* is shown in dark purple. Islets are not drawn to scale. (E) Maximum likelihood reconstruction of phylogeny based on 63,994 core SNPs among 41 *S.* Javiana isolates and a representative isolate of *S.* Mississippi (used to root the phylogeny). The *S.* Javiana strain (FSL S5-0395) used in phenotypic experiments is shown in bold. Circles at nodes represent bootstrap values >90 (based on output from 1,000 bootstrap repetitions). Assembly names are colored to reflect the country from which the isolate originated; the isolation sources (human clinical shown in orange, environmental shown in yellow) as well as the nucleotide identity of TT genes (light blue) and *artB* (purple) are shown as colored bars. Roman numerals are used to depict the clades of the *S.* Javiana isolates.

Multiple studies have suggested that genes encoding the TT (*cdtB*, *pltA*, and *pltB*) are distributed across multiple lineages and serovars of *Salmonella* (27–29). To better understand the evolution of the TT, we queried whole-genome sequence (WGS) data for representative isolates of 235 unique S. enterica subsp. *enterica* serovars to assess the distribution of TT genes across the main lineages of S. enterica (29). All three TT genes were detected in 105 S. enterica subsp. *enterica* serovars (45% of serovars examined here) (Fig. 1A). *cdtB*, encoding one of the active subunits, was detected in an additional seven S. enterica subsp. *enterica* serovars as well as in representative strains of S. enterica subsp. *arizonae* and S. enterica subsp. *diarizonae* (Fig. 1A). While *pltA* and *pltB* were not detected in these seven serovars or the representative S. enterica subsp. *arizonae* and S. enterica subsp. *diarizonae* strains with the blast settings used, visual examination of these genomes suggested that all except *S.* Napoli encoded open reading frames (ORFs) that showed nucleotide sequence similarity to *S.* Javiana *pltA* (79% to 82% identity) and *pltB* (65% to 68% identity) (see Table S1 in the supplemental material). Therefore, these serovars/strains were regarded as encoding putative *pltA* and *pltB* but were not considered encoding all TT genes for the analyses reported below. Proportions of serovars encoding all TT genes were significantly different among S. enterica phylogenetic clades (*P* < 0.001; Fisher’s exact test); clade B had the highest proportion of TT-encoding serovars (80 of 83 serovars), followed by section Typhi (14 of 21 serovars), while TT genes were detected in significantly fewer clade A1 and A2 serovars (*P* < 0.001 for all pairwise comparisons) with just 17% (8 of 40 serovars) and 4% (3 of 76 serovars) of serovars in these clades encoding TT genes, respectively (Fig. 1B). These results demonstrate a strong phylogenetic relationship between the presence of TT genes and *Salmonella* clades.

10.1128/mSphere.01255-20.3 TABLE S1 Nucleotide lengths and identities of putative *pltA* and *pltB* that were not detected with initial blast searches. Download Table S1, PDF file, 0.1 MB. Copyright © 2021 Gaballa et al. 2021 Gaballa et al. This content is distributed under the terms of the Creative Commons Attribution 4.0 International license.

Next, we assessed the genomic location of the TT islet using WGS data for representative serovars (see Materials and Methods for a description of how these were selected) from each TT-positive S. enterica subsp. *enterica* clade that had a closed genome available. The TT islet was previously reported to be located within *Salmonella* pathogenicity island (SPI) 11 (5, 28); however, the genomic location of SPI-11, and therefore the TT genes, was unknown. While the size of SPI-11 was variable among the serovars examined here, the TT islet was consistently located approximately 3 kb from the terminus of SPI-11. For all genomes evaluated (representing S. enterica serovar Goldcoast [clade A1], S. enterica serovar Milwaukee [clade A2], S. enterica serovar Paratyphi A [section Typhi], S. enterica serovar Indiana [section Typhi], S. enterica serovar Poona [clade B], and *S.* Javiana [clade B]), except *S*. Typhi, SPI-11 was within 5 to 7 kb from *phoP-phoQ*, which encodes a two-component regulatory system that has been shown previously to be the primary system regulating transcription of TT genes (30). However, in *S.* Typhi CT18, SPI-11 was located >550 kb upstream of *phoP-phoQ*. These data suggest that, in general, both the location of SPI-11 and the location of the TT islet within SPI-11 are relatively conserved.

While the overall phylogeny suggests multiple acquisition and/or deletion events of TT genes, the most likely scenario appears to be that TT genes were present in the S. enterica ancestor, and subsequent deletion events in major clades as well as possibly additional independent (re)acquisitions of TT genes (e.g., in some serovars in clade A1 and A2 and section Typhi) occurred.

### The majority of TT-encoding serovars also encode *artB*, which is located on an islet within an operon encoding genes associated with resistance to antimicrobial peptides.

Several groups previously established that multiple TT-encoding serovars also encode a homologous binding subunit, *artB* (28, 31, 32). Therefore, we also assessed the presence of *artB* to determine the frequency that TT and ArtB are coencoded in a given serovar. Among all 235 serovars examined, *artB* was detected in a total of 88 serovars (Fig. 1A). Proportions of *artB-*encoding serovars were significantly different across the phylogenetic clades (*P* < 0.001; Fisher’s exact test) (Fig. 1C). Serovars in clades B (66 of 83) and D (3 of 3) and section Typhi (12 of 21) had significantly higher proportions of *artB*-positive serovars than clade A1 (4 of 48) and A2 serovars (3 of 79) (see Fig. 1C for false-discovery rate [FDR] corrected *P* values for pairwise comparisons). Among the 105 serovars for which a full TT islet was detected (i.e., excluding the three clade D serovars, which encoded putative *pltA* and *pltB*), *artB* was detected in 85 (i.e., 81% of TT-encoding serovars also encoded *artB*).

Given the relative conservation of *artB* among TT-positive serovars, we next assessed whether the location of the *artB* islet was conserved among genomes representing the different S. enterica subsp. *enterica* phylogenetic clades (A1, A2, and B and section Typhi). Although the genomic locations and the numbers of ORFs identified differed slightly (Fig. 1D; see also Table S2), we found that in all cases, the *artB* islet was inserted between *sapB* and *sapC* (Fig. 1D). The GC content of the *artB* islet was considerably lower (average, 42.2%; range, 41.2% to 44.7%) than the genome-wide GC content (average, 52.2%; range, 52.1% to 52.3%), suggesting that this islet was likely acquired via horizontal transfer from another source (Table S2). In addition to *artB* itself, the *artB* islet includes (i) between 4 to 7 ORFs with no known or predicted functions (Table S2) and (ii) an *artA* pseudogene, which was identified in all islets examined. The *artA* pseudogene contains multiple mutations leading to premature stop codons, with the most 5′ premature stop codon leading to a predicted peptide with just 4 amino acid (aa) residues. For one serovar, *S.* Milwaukee, an additional ORF annotated as an IS*3* family transposase, was detected in the middle of the *artB* islet. Together, these results suggested a common site-specific mode of acquisition of the *artB* islet as well as a selective advantage for maintenance and/or acquisition of the *artB* islet among most TT-positive serovars.

10.1128/mSphere.01255-20.4 TABLE S2 Positions of *artB* islet in closed genomes from representative strains of TT and *artB*-positive serovars. Download Table S2, PDF file, 0.1 MB. Copyright © 2021 Gaballa et al. 2021 Gaballa et al. This content is distributed under the terms of the Creative Commons Attribution 4.0 International license.

### For serovar *S.* Javiana, TT genes are highly conserved, while the presence of intact *artB* is clade dependent.

Given that 96% of clade B serovars examined here were TT positive and the majority of studies examining the TT had been conducted with *S.* Typhi, we characterized the role of *artB* in the clade B nontyphoidal serovar *S.* Javiana, which is the most prevalent TT-positive serovar among human clinical salmonellosis cases in the United States (33). Among a collection of 41 *S.* Javiana isolates selected to represent a range of genotypes (different single nucleotide polymorphism [SNP] clusters), geographical sources, and dates of isolation (see Table S3), we found that sequences of *cdtB*, *pltA*, and *pltB* were highly conserved (99% to 100% nucleotide identity). In contrast, while *artB* was conserved for most of the isolates (100% identity for 34/41 isolates; clade III) (Fig. 1E), we also detected two small clades for which *artB* was more variable, including (i) five isolates encoding an *artB* allele containing a 46-nucleotide deletion resulting in a premature stop codon (clade I) (Fig. 1E), and (ii) two isolates for which *artB* was not detected with blast (clade II) (Fig. 1E); visual inspection of the *sap* operon further confirmed the absence of the *artB* islet at this locus as well as a lack of raw sequence reads mapping to *artB*. These results suggested that even though *artB* is also relatively conserved among *S.* Javiana isolates, loss of ArtB function occurred independently in two clades of *S.* Javiana, due to either *artB* deletion or *artB* pseudogene formation.

10.1128/mSphere.01255-20.5 TABLE S3 SRR run identity and metadata for 41 *S.* Javiana isolates and one *S.* Mississippi isolate used for phylogenetic and blast analyses. Download Table S3, PDF file, 0.1 MB. Copyright © 2021 Gaballa et al. 2021 Gaballa et al. This content is distributed under the terms of the Creative Commons Attribution 4.0 International license.

### *artB* and *pltB* are induced when *S.* Javiana is grown under nutrient-limiting conditions, such as those representing an intracellular host environment.

**FIG 2:**
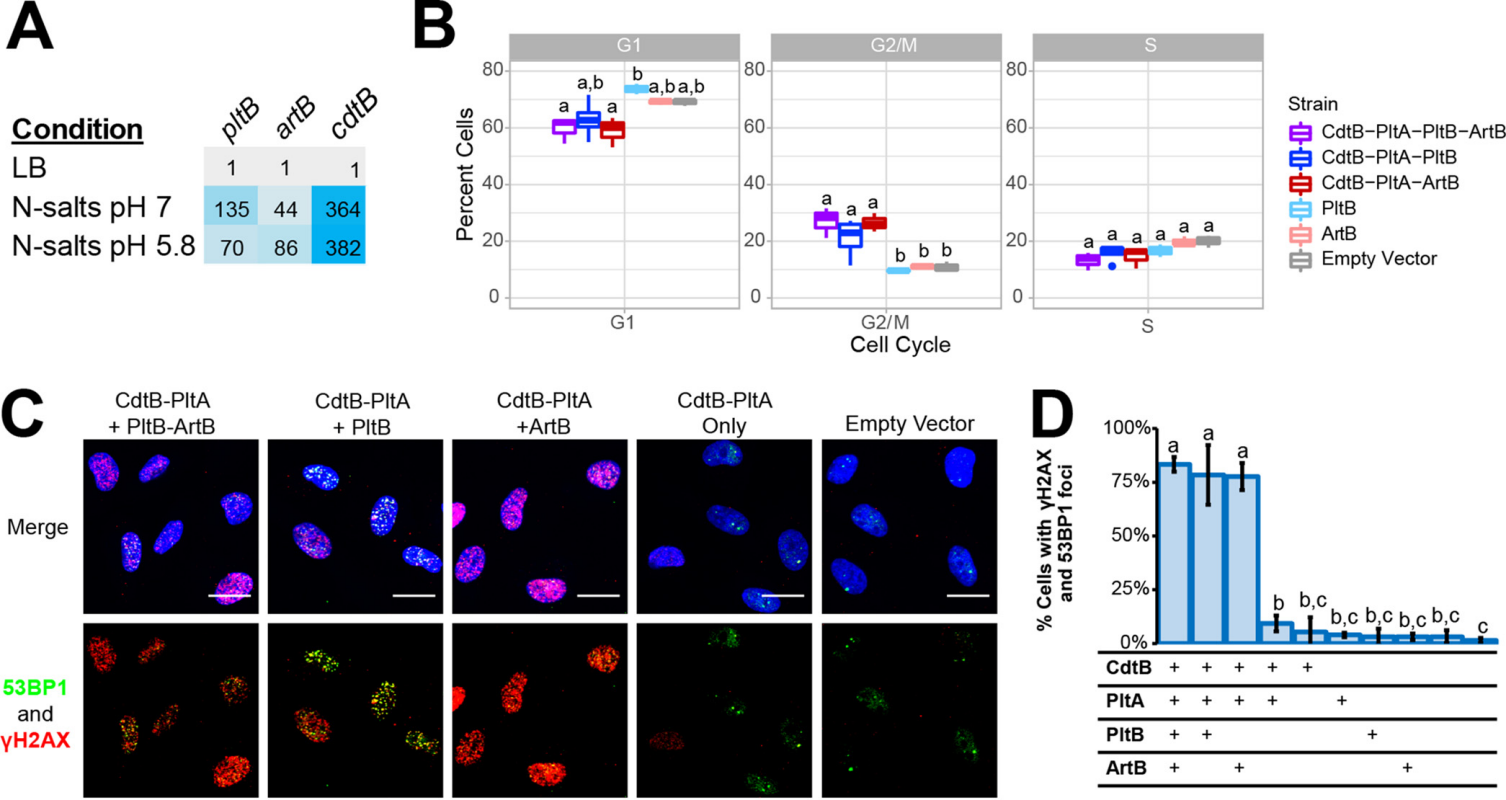
ArtB or PltB can be used to form an active TT binding subunit. (A) Fold expression (2^−ΔΔ^*^CT^*) of *pltB*, *cdtB*, and *artB* in *S.* Javiana cells grown for 5 h in either N-salts minimal medium at pH 7 or pH 5.8 (representing mid-exponential phase for this condition), normalized to expression of *S.* Javiana cells grown for 3 h in LB broth, pH 7 (representing mid-exponential phase for this condition). Results are averaged from three independent experiments; cells are shaded to represent high (blue) and low expression (gray) relative to expression in LB broth. (B) Cell cycle analyses of HIEC-6 cells coincubated with lysates of E. coli BTH101 cells expressing different combinations of tagged typhoid toxin subunits: CdtB-His, PltA-Strep, PltB-3×-Flag, and ArtB-c-Myc. HIEC-6 cells were coincubated with toxins for 24 h prior to analyzing DNA content with propidium iodide staining and analysis by flow cytometry. Data represent the averages from three independent experiments; boxplots that do not share lowercase letters are statistically different (Tukey’s adjusted *P* value < 0.05). (C) Assessment of DNA damage response activation among HIEC-6 cells treated with toxin subunits. Immunofluorescence staining of HIEC-6 cells coincubated for 24 h with total cell lysates containing different combinations of CdtB-His, PltA-Strep, PltB-3×-Flag, and ArtB-c-Myc. DNA damage response proteins are shown in green (53BP1) and red (γH2AX); nucleic acids were stained with DAPI (blue). Scale bars, 20 µm. (D) Quantification of HIEC-6 cell nuclei having ≥4 53BP1-foci as well as γH2AX foci; the table below the graph shows which subunits were present in the supernatant added to the HIEC-6 cells. Two negative controls were included and are represented by the final two bars in the graphic, which represent HIEC-6 cell populations that were treated with supernatants from E. coli BTH101 cells with an empty vector (left) and untreated cell populations (right). Lowercase letters indicate statistically significant differences in the proportions of cells having DDR foci; bars that share lowercase letters are not significantly different at α = 0.05 after correcting for multiple comparisons.

Expression of *pltB* in *S.* Typhi was previously shown to occur when cells were grown under conditions that mimic those encountered by intracellular *Salmonella* (30, 32). Therefore, we compared *pltB*, *cdtB*, and *artB* transcript abundances of *S.* Javiana grown in Luria-Bertani (LB) broth (pH 7) and N-salts minimal medium containing 8 µM Mg^2+^ (pH 7). When *S.* Javiana was grown in N-salts minimal medium at pH 7, transcript abundances of *pltB*, *cdtB*, and *artB* were on average 135-, 364-, and 44-fold higher, respectively, than abundances in *S.* Javiana grown in LB broth (Fig. 2A). When *S.* Javiana was grown in N-salts minimal medium acidified to pH 5.8 to simulate intracellular conditions (34), transcript abundances of *cdtB* and *artB* were higher (on average 382- and 86-fold compared to growth in LB broth) (Fig. 2A), while *pltB* transcript abundances were approximately 2-fold lower than abundances obtained during growth in N-minimal medium at pH 7. Collectively, these results suggested that *artB* and *pltB* transcription is induced similarly to that of *cdtB* when *S.* Javiana is grown in minimal medium where Mg^2+^ is limiting. However, the relative transcript abundances (relative to growth in LB broth) of *pltB* were higher at neutral pH than at acidic pH, while both *cdtB* and *artB* had higher relative transcript abundances at acidic pH, suggesting that pH may serve as an environmental cue for modulating expression of *pltB* and *artB*.

### Binding subunits composed of PltB or ArtB result in active TT.

Previous gene knockout experiments suggested that *S.* Javiana requires either ArtB or PltB to produce active TT, as strains with deletion of either *artB* or *pltB* still showed production of active toxin (16). To biochemically confirm this, we expressed various combinations of toxin subunits in a heterologous host and then coincubated lysates containing these toxins with human intestinal epithelial cells (HIEC-6) to assess toxin activity. The accumulation of cells in the G_2_/M phase continues to be the gold standard for assessing TT activity (14, 17), as cells that have an activated DNA damage response (DDR) will accumulate at this cell cycle checkpoint (35). Therefore, we analyzed the cell cycle progression of HIEC-6 cells treated with lysates containing holotoxins with PltB or ArtB to confirm the activity of both toxin types. Treatment of HIEC-6 cells with lysates containing CdtB, PltA, and either PltB or ArtB resulted in a significantly higher proportion of HIEC-6 cells in the G_2_/M phase (PltB as binding subunit, *P* = 0.037, or ArtB as binding subunit, *P* = 0.006; both compared to cells treated with lysates containing empty vector) (Fig. 2B). There was no significant difference in the proportion of cells in the G_2_/M phase for HIEC-6 cells treated with TT that used ArtB versus PltB as the binding subunit (*P* = 0.6983), supporting that holotoxins using either monomer for the binding subunit are active. To confirm that the accumulation of cells in G_2_/M phase after treatment with these toxins was due to the activation of a DDR, we also used immunofluorescence (IF) staining to detect two proteins involved in the DDR, namely, phosphorylated histone H2AX (γH2AX), and 53 binding protein 1 (53BP1). Treatment with lysates containing CdtB, PltA, and either PltB or ArtB as the binding subunit resulted in DDR activation in ∼78% of HIEC-6 cells (Fig. 2C and D), confirming that both holotoxins are active. Importantly, treatment with individual toxin subunits did not result in DDR activation among treated cells (Fig. 2C and D). Together, these data confirm that the TT encoded by *S.* Javiana can use either PltB or ArtB as the binding subunit.

### *In silico* modeling suggests that heteropentameric binding subunits of ArtB and PltB may occur.

**FIG 3:**
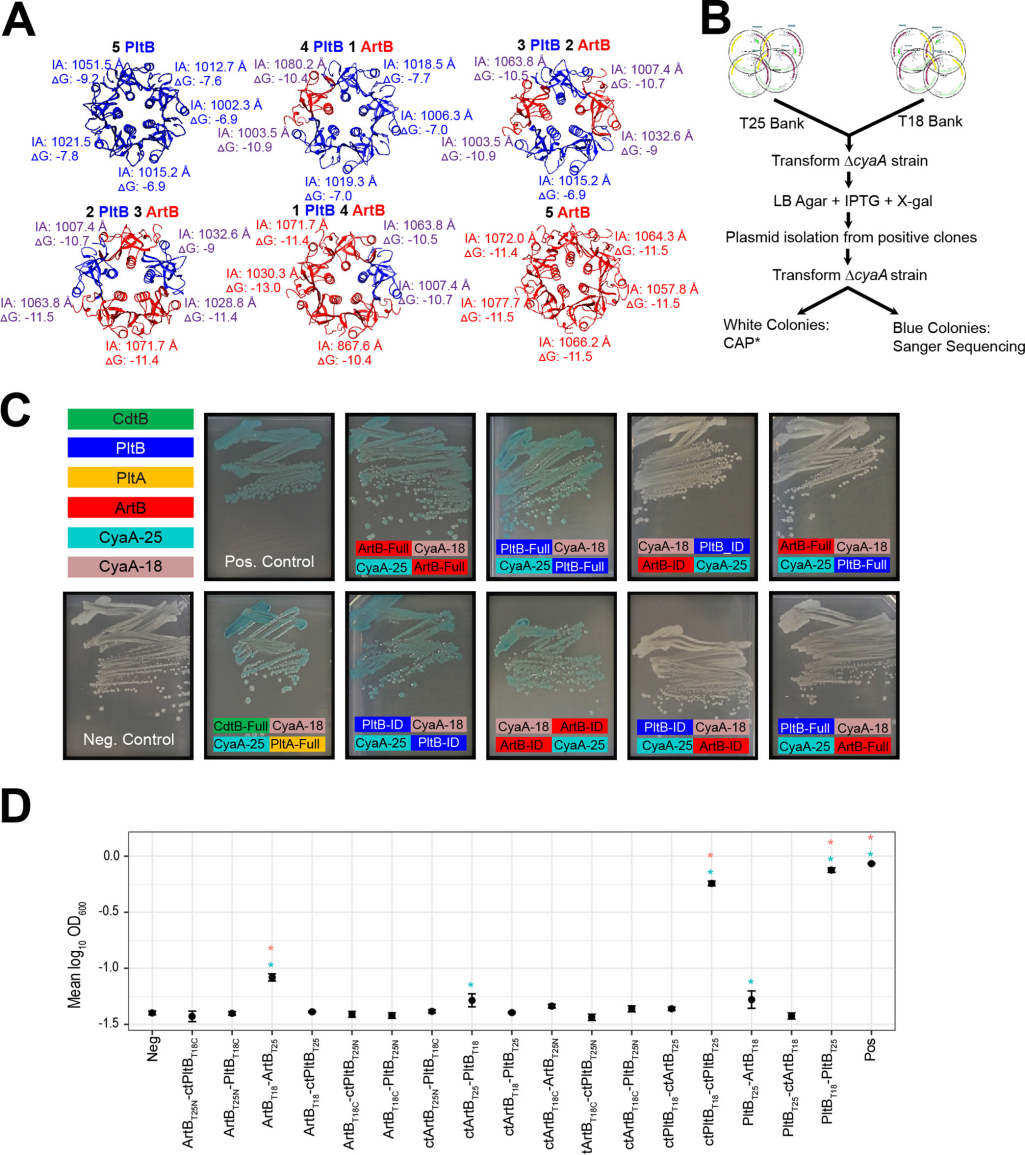
Analysis of PltB-ArtB interactions. (A) Solvation energies of the interface calculated between monomers in the binding subunit are color coded to represent the interaction: PltB-PltB are shown in blue, ArtB-ArtB in red, and PltB-ArtB in purple. IA, interface area in Angstroms (Å); Δ*G*, solvation free energy gain upon formation of the interface (kcal/M). Negative Δ*G* values correspond to hydrophobic interfaces, and therefore a positive protein affinity. (B) Schematic of the method used to screen two-hybrid system clones. In the example shown, DNA fragments encoding the PltB polypeptide or C-terminal oligomerization region, as predicted from TT structure, were cloned at the 5′ or 3′ ends of regions encoding adenylate cyclase domain T18 or T25. All T18 or T25 clones were pooled into “banks” that were cotransformed into the cAMP-null (Δ*cyaA*) E. coli BTH101. Screening was performed by growing the transformants on LB agar supplemented with IPTG and X-Gal. Plasmid DNA was isolated from clones with positive interactions and was retransformed into E. coli BTH101 to exclude constitutively active CAP* mutants. Clones with confirmed interactions were submitted for Sanger sequencing to determine the identity of the interacting domains. (C) E. coli BTH101 two-hybrid system strains grown on LB agar supplemented with IPTG and X-Gal. Clones that showed positive interactions appear blue. Colored boxes show the fusion of either the interacting domain (_ID) or the full-length protein (_Full) for PltB, PltA, CdtB, and ArtB to either the CyaA-25 (shown in teal) or CyaA-18 subunits (shown in mauve) of the two-hybrid system. (D) Detection of interactions of ArtB and PltB in *S.* Javiana Δ*cyaA* harboring two-hybrid system plasmids. *S.* Javiana cells were grown for 24 h in M63 minimal medium with maltose as the sole carbon source, and absorbance was measured at 600 nm. Error bars indicate the standard deviations of the means from three independent experiments. Constructs with the C terminus are denoted with “ct.” *, *P* < 0.05 versus the negative control before (blue) and after (pink) Dunnett’s test for multiple comparisons adjustment; the PltB_T25_-ArtB_T18_ interaction was only marginally significant after multiple comparison corrections (corrected *P* value = 0.0973).

Given that both ArtB and PltB can form active TT and that transcript abundances of *artB* and *pltB* are significantly higher when *S.* Javiana is grown under nutrient-limiting conditions, such as those that might be encountered by intracellular *Salmonella*, we hypothesized that these subunits likely compete for inclusion in the TT, resulting in toxin binding subunits composed of homo- and heteropentamers. The structures of the TT binding subunits containing homopentamers of PltB (from *S.* Typhi [18]) or ArtB (from *S.* Typhimurium DT104 [26]) have been resolved experimentally and were used here to model and predict the energies of interactions between monomers in binding subunits composed of various ratios of ArtB and PltB using the predominant amino acid sequence types for *S.* Javiana (Fig. 1E and 3A). While alignment of ArtB from *S.* Typhimurium DT104 with ArtB from clade III *S.* Javiana (Fig. 1E) showed only 74% amino acid identity, interface areas and the Δ*G* solvation energy gain of the ArtB complex formation for *S.* Typhimurium DT104 ArtB and *S.* Javiana ArtB were similar (see Fig. S1), supporting the modeling strategy used. ArtB was shown to interact with more specificity and stronger hydrophobicity with itself (ArtB-ArtB Δ*G* average, −11.5 kcal/M) than with PltB (ArtB-PltB Δ*G* average, −10.5 kcal/M) (Fig. 3A); however, the theoretical interaction of ArtB-PltB was predicted to be more favorable than PltB-PltB (Δ*G* average, −7.5 kcal/M) (Fig. 3A). These data suggested that ArtB and PltB monomers may interact to form heteropentameric binding subunits, which we next tested experimentally.

10.1128/mSphere.01255-20.1 FIG S1 Results of additional modeling and two-hybrid system constructs in E. coli BTH101 cells. (A) Modeling was performed to compare the calculated energies for *S.* Typhimurium DT104 ArtB (shown in red font) and *S.* Javiana CFSAN001992 ArtB (shown in black font). (B) Additional two-hybrid system clones tested as in Fig. 3C. Briefly, E. coli BTH101 cells were transformed with plasmids encoding partial interacting domain (_ID) or full-length proteins (_Full) fused to T18 and T25 subunits of CyaA (50). Blue pigmentation suggests an interaction, while tan colonies are unable to confirm an interaction. Some of these constructs are also shown in Fig. 3C and were included again here for convenience (i.e., showing the entire plate or half of the plate). Download FIG S1, PDF file, 2.8 MB. Copyright © 2021 Gaballa et al. 2021 Gaballa et al. This content is distributed under the terms of the Creative Commons Attribution 4.0 International license.

### *In vitro* interactions between ArtB and PltB monomers and copurification of ArtB and PltB from TTs suggest that heteropentameric binding subunits may occur *in vitro*.

We first assessed the possibility of ArtB-PltB interactions using an adenylate cyclase two-hybrid system in Escherichia coli BTH101. We specifically used a high-throughput screening approach (Fig. 3B) to test for possible interactions of different domains for each toxin component (CdtB, PltA, PltB, and ArtB) including (i) full-length proteins, (ii) polypeptides without the signal peptide sequence, and (iii) the oligomerization domains predicted from the TT structure (18). Using this technique, we confirmed PltB-PltB, ArtB-ArtB, and CdtB-PltA interactions for both interacting domains and full-length toxin subunits (Fig. 3C), but we were unable to confirm a PltB-ArtB interaction, despite testing interactions for various combinations of interacting domains and full-length PltB and ArtB (Fig. 3C and Fig. S1).

In a heterologous host two-hybrid system assay, issues arising from protein fusion stability and folding as well as a requirement of basal levels of cAMP to initiate the positive-feedback induction loop may interfere with the ability of the system to detect some protein-protein interactions. Hence, we also constructed a cAMP-null *S.* Javiana strain (Δ*cyaA*) to assess possible interactions in *Salmonella*. When Δ*cyaA S.* Javiana was grown in M63 broth (supplemented with 0.02 mM cAMP, a level that induced basal fusion protein expression but did not support growth of negative controls), we found marginally significant (*P* < 0.05, uncorrected *P* value) PltB-PltB, ArtB-ArtB, and ArtB-PltB interactions (Fig. 3D and Table S4); however, upon correcting for multiple comparisons, only one of the ArtB-PltB interactions was considered statistically significant at an α of 0.1 (corrected *P* value = 0.0973) (Fig. 3D and Table S4). These results suggested that while PltB-PltB and ArtB-ArtB interactions were more readily detected using the two-hybrid system, there was some evidence of ArtB-PltB interactions; we therefore conducted additional experiments to determine whether both ArtB and PltB subunits could be incorporated into a heteropentameric configuration of the TT binding subunit.

10.1128/mSphere.01255-20.6 TABLE S4 Estimates of linear model on log10-transformed absorbance (OD_600_) data of *S*. Javiana Δ*cyaA* mutants. Download Table S4, PDF file, 0.09 MB. Copyright © 2021 Gaballa et al. 2021 Gaballa et al. This content is distributed under the terms of the Creative Commons Attribution 4.0 International license.

**FIG 4:**
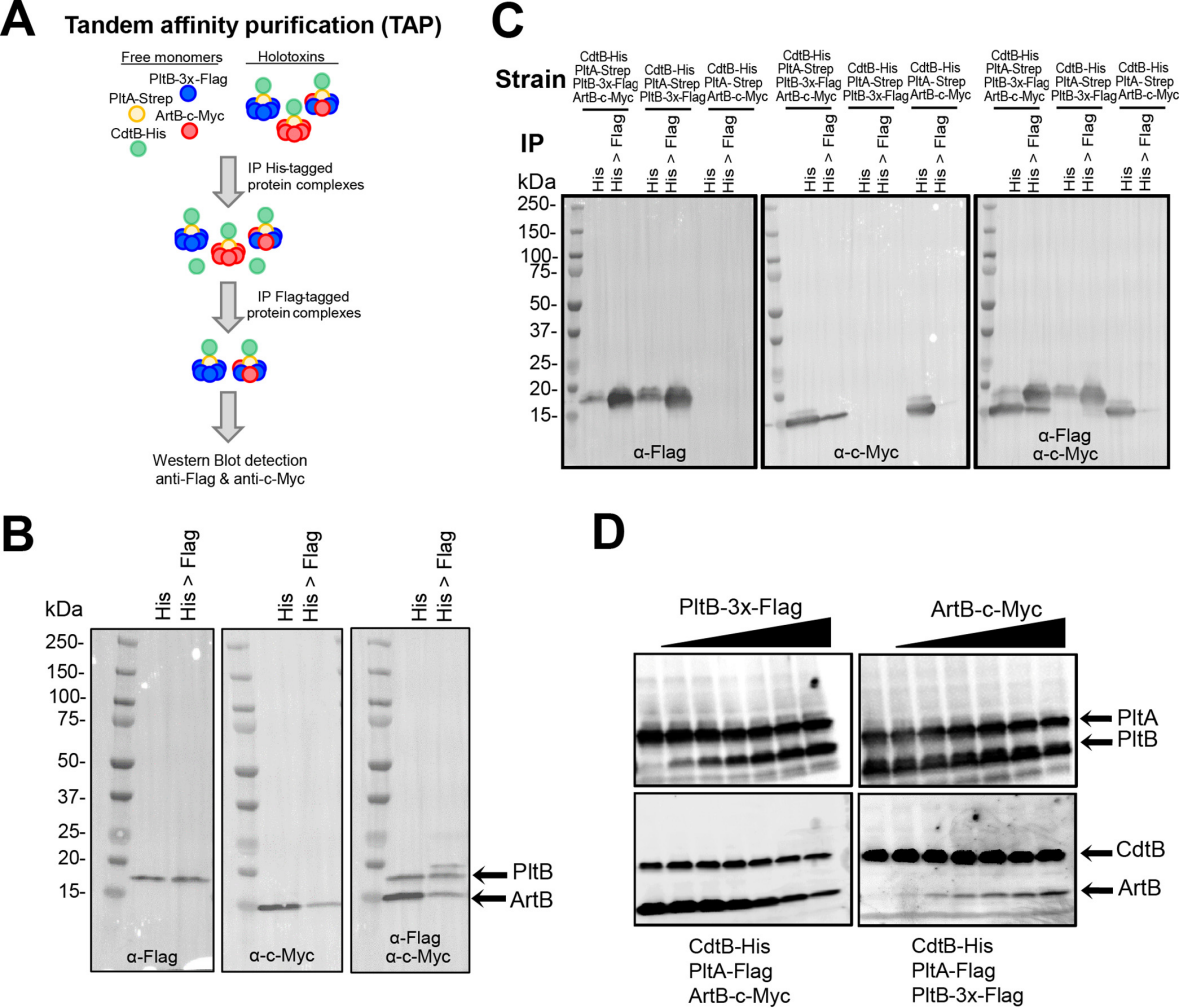
PltB replaces ArtB for inclusion in the holotoxin. (A) Schematic for the approach used for TAP and Western blot detection of heteropentameric binding subunits. (B) Detection of PltB-3×-Flag and ArtB-c-Myc using TAP and Western blotting. Holotoxins were pulled down from E. coli BTH101 strains expressing CdtB-His, PltA-Strep, PltB-3×-Flag, and ArtB-c-Myc using α-His antibodies (His) followed by purification of PltB-3×-Flag-containing holotoxins by pulling down with α-Flag magnetic beads (lane His>Flag). Proteins were visualized with antibody staining to detect PltB-3×-Flag (left), ArtB-c-Myc (middle), and both PltB-3×-Flag and ArtB-c-Myc (right). Results of additional replicates can be found in Fig. S2 in the supplemental material. (C) Immunoblots demonstrating the specificity of the pulldown assays and antibody detection used for TAP (additional blots for additional replicates can be found in Fig. S2). (D) Results of the binding subunit exchange assay. PltB-3×-Flag was added at various concentrations to lysates containing CdtB-His, PltA-Flag, and ArtB-c-Myc (left), or ArtB-c-Myc was added to lysates containing CdtB-His, PltA-Flag, and PltB-3×-Flag (right). Holotoxins were purified by pulling down with α-His antibodies to detect CdtB-His, and subunits were detected using tag-specific antibodies (PltA-Flag, CdtB-His, and PltB-3×-Flag). Results from one representative experiment are shown; the assay was performed as three independent experiments. Images for additional replicates can be found in Fig. S2.

Previous studies have suggested that the TT binding subunit exists as a stable pentamer (18). Therefore, we also used tandem affinity purification (TAP) of different combinations of CdtB-His, PltA-Strep, PltB-3×-Flag, and ArtB-c-Myc overexpressed in E. coli BTH101 (Fig. 4A) to assess whether holotoxins can be formed with heteropentameric binding subunits of PltB and ArtB. Purification of His-tagged CdtB followed by purification of PltB-3×-Flag (Fig. 4B) revealed that ArtB was consistently (results of three independent experiments) (see Fig. S2) copurified with PltB, albeit at lower levels than PltB, in both pulldown steps, supporting that PltB and ArtB can form heteropentameric binding subunits. Additional immunoblotting to control for the possibility of recognition of ArtB-c-Myc by anti-Flag antibodies (used to detect PltB-3×-Flag) confirmed the specificity of this approach (Fig. 4C and Fig. S2). Taken together, both the weak interaction detected using the two-hybrid system and the copurification of ArtB-c-Myc from PltB-3×-Flag-containing toxins suggested that binding subunits composed of heteropentamers of ArtB and PltB may occur, further supporting a scenario in which ArtB and PltB monomers may compete for inclusion in the TT binding subunit.

10.1128/mSphere.01255-20.2 FIG S2 Additional biological replicates and controls for experiments shown in Fig. 4. (A) Detection of ArtB-c-Myc with rabbit α-c-Myc antibodies following immunoprecipitation with α-His (lane His) or α-His and α-Flag (lane His>Flag) antibodies from whole-cell lysates containing various combinations of TT proteins and ArtB. (B) Biological replicates 2 and 3 for Fig. 4B in the main text. Immunoprecipitation of lysates from E. coli BTH101 expressing CdtB-His, PltA-Flag, PltB-3×-Flag, and ArtB-c-Myc was performed using either α-His (lane His) or α-His and α-Flag antibodies (lane His>Flag), followed by detection of ArtB-c-Myc (lower band) and PltB-3×-Flag (upper band). (C) Additional replicates 2 and 3 for Fig. 4D in the main text. PltB-3×-Flag was added at various concentrations to lysates containing CdtB-His, PltA-Flag, and ArtB-c-Myc (left), or ArtB-c-Myc was added to lysates containing CdtB-His, PltA-Flag, and PltB-3×-Flag (right). Holotoxins were purified by pulling down with α-His antibodies to detect CdtB-His, and subunits were detected using tag-specific antibodies (PltA-Flag, CdtB-His, PltB-3×-Flag, and ArtB-c-Myc). For replicate number 2, as well as replicate number 1 shown in Fig. 4D in the main text, the eluent from pulldown assays representing each level of added PltB-3×-Flag or ArtB-c-Myc was divided into two aliquots, which were then electrophoresed on separate gels and detected on separate blots to enable better resolution of PltA and PltB; for replicate no. 3, the eluent for each set of pulldowns (i.e., with different amounts of added PltB-3×-Flag [left] or ArtB-c-Myc [right]) was run on a single gel, and all three antibodies (α-His, α-Flag, and α-c-Myc antibodies) were added to the same membrane for detection of CdtB-His, PltA-Flag, PltB-3×-Flag, and ArtB-c-Myc. Download FIG S2, PDF file, 1.8 MB. Copyright © 2021 Gaballa et al. 2021 Gaballa et al. This content is distributed under the terms of the Creative Commons Attribution 4.0 International license.

### PltB and ArtB may compete for inclusion in the holotoxin.

Collectively, our data suggested that *artB* and *pltB* are both induced when *Salmonella* is grown in medium mimicking intracellular conditions during *Salmonella* infection of epithelial cells, and both ArtB and PltB can be used to form biologically active toxins. Therefore, we hypothesized that ArtB and PltB monomers likely compete for inclusion in the holotoxin binding subunit. We thus used a modified competition assay to assess whether addition of PltB to lysates containing CdtB, PltA, and ArtB resulted in replacement of ArtB in the holotoxin, and vice versa. Cell lysate from E. coli BTH101 overexpressing CdtB, PltA, and either ArtB or PltB was challenged with various concentrations of PltB- or ArtB-containing lysate, respectively, prior to TAP and subsequent Western blot detection. Upon challenging cell lysates containing CdtB, PltA, and ArtB with increasing amounts of lysates containing PltB, we found that PltB replaced ArtB, thereby reducing the total amount of ArtB bound in the holotoxin (Fig. 4D and Fig. S2). This result was not reciprocal, however, as addition of ArtB only marginally reduced the amount of PltB in the holotoxin (Fig. 4D and Fig. S2). Together, this suggests that PltB and ArtB may compete for inclusion in the binding subunit.

## DISCUSSION

Multiple investigations into the role of ArtB, a homolog of PltB, have suggested that ArtB can form a functional TT binding subunit (16, 26, 32). Our results suggest a role for ArtB in the diversification of the TT in NTS serovars, as (i) both toxin subunits are coencoded by serovars spanning multiple lineages of S. enterica subsp. *enterica*, (ii) both *artB* and *pltB* are expressed under conditions mimicking the intracellular environment, with differences in transcript abundances at neutral and acidic pH potentially favoring assembly of TT with different binding subunits (ArtB or PltB) under different environmental conditions, and (iii) ArtB and PltB may compete for inclusion in the binding subunit.

### TT genes are considerably more widespread among S. enterica subsp. *enterica* serovars than previously thought.

Owing to the initial discovery of *cdtB* in *S.* Typhi strain CT18 in 2000, the majority of research efforts have, and continue to, focus primarily on the potential contributions of this toxin to typhoid fever (17, 18, 24, 30, 32, 36–40). Several studies have, however, demonstrated that TT is encoded by many nontyphoidal serovars (27–29, 31), suggesting that this toxin is not the sole contributing factor to the acute onset of typhoid fever, an observation which was recently confirmed in a human challenge study that demonstrated that onset of acute typhoid fever occurred even in subjects challenged with a TT-null strain (24). Importantly, our results support that TT genes are common in clade B serovars and are rare among clade A1 and A2 serovars. As the number of WGS data available for NTS serovars continues to increase, the number of serovars that are known to encode TT will likely increase. Regardless, our results suggest that studies examining the role of the TT in NTS serovars are warranted given the conservation of this toxin in nearly all clade B serovars, as reported here and in previous studies (28, 29).

### Maintenance of the *artB* islet in TT-encoding serovars representing multiple lineages of S. enterica subsp. *enterica* suggests an important role for ArtB.

Previous studies had suggested that some serovars encode both TT genes and *artB* (27, 31, 32). Our analyses demonstrate that 81% of TT-positive serovars also encode *artB*. More importantly, our results indicate that the location of the *artB* islet within the *sap* operon is consistent across representative serovars in clades A1, A2, and B and section Typhi. *sap* operon genes have been shown to play a role in resistance to host-derived antimicrobial peptides for *S.* Typhimurium (41), uropathogenic E. coli (42), and Haemophilus ducreyi (43). In 2010, Rodas et al. demonstrated that integration of the genomic islet GICT18/1 (putative *artB* islet) into *S.* Typhi *sapB* resulted in an increased sensitivity to the antimicrobial peptide protamine (44). Furthermore, the authors showed that excision of GICT18/1 restored transcription of *sapD* and *sapF* and restored resistance to protamine to levels similar to that observed for *S.* Typhimurium, which lacks the GICT18/1 islet (44). As insertion of the *artB* islet is expected to result in sensitization to antimicrobial peptides, these results suggest that there is likely an advantage for the selective maintenance of *artB* by most TT-encoding serovars.

This begs the question of why the majority of TT-positive *Salmonella* serovars would evolve to encode multiple binding subunits, especially if acquisition of one (ArtB) may result in an increased susceptibility to host-derived antimicrobial peptides. Our current understanding is that PltB and ArtB have adapted to bind to different cell surfaces (26, 32), thereby expanding the variety of cell types that TT can bind to. While PltB has been reported to preferentially bind Neu5Ac-terminated glycans (26), ArtB can bind both Neu5Ac- and Neu5Gc-terminated glycans (26), suggesting that PltB and ArtB allow TT to bind to different cell types. Furthermore, Lee et al. recently established that specific amino acid residues in PltB produced by *S.* Javiana (called Javiana toxin in that study) were associated with toxin binding to intestinal epithelial cells but not to endothelial cells of brain arterioles (45), therefore supporting the idea that PltB produced by *S.* Typhi and *S.* Javiana may have evolved to enable binding to different tissues. Given that single amino acid changes have been found to significantly dampen binding affinity to cell surface glycans (45), collectively, this suggests that encoding two different subunits may provide an evolutionary advantage, as it likely enables TT-positive *Salmonella* to affect a wider range of cell types, tissues, and possibly hosts. Therefore, future studies examining the sequence variants of ArtB and PltB among a broader range of *Salmonella* serovars, as well as the binding affinities for these variants for a panel of different cells representing different tissues and hosts, will provide a more complete picture of the potential role of the TT binding subunit in facilitating tissue and or host tropism.

Furthermore, there is also some evidence to support the role of TT as an immunotoxin. Previous reports showed that injection of TT containing PltB or ArtB (called PltC in *S.* Typhi) into C57BL/6 mice resulted in significantly lower levels of total white blood cells, lymphocytes, and monocytes, than injection with phosphate-buffered saline (PBS) (32). In a human challenge study, subjects that were administered TT-null *S.* Typhi had higher fold changes of IgA and IgG against O9:lipopolysaccharide (LPS) than subjects that were administered wild-type TT (although not explicitly addressed in the study, this strain would also encode *artB*) (24). Therefore, TT may play a role in dampening the adaptive immune response, which may have implications for reinfection with TT-producing *Salmonella*. While these studies suggest that TTs containing ArtB and/or PltB may act as an immunotoxin, additional experimentation to assess the expression of and activity of ArtB- and PltB-containing TTs will be essential for addressing TT’s potential role in modulating the immune response during infection with typhoidal and nontyphoidal TT-producing serovars.

### While copurification of ArtB and PltB suggest the formation of heteropentameric forms of the TT binding subunit, homopentamers appear to be the predominant form of the binding subunit.

Overall, our data, combined with previous studies, suggest that multiple mechanisms likely fine-tune ArtB and PltB expression and their assembly into different TT forms (i.e., toxins having binding subunits containing just PltB or ArtB or a mix of both PltB and ArtB). At the transcriptional level, both *artB* and *pltB* are induced when cells are grown under low-Mg^2+^ conditions (this study and reference 30). However, our results also indicate that pH may serve as an additional environmental cue modulating expression of *artB* and TT genes. For example, *artB* is more highly induced under acidified conditions (pH 5.8), while induction of *pltB* is higher at neutral pH. In *S.* Typhi, *artB* and *pltB* are regulated by different two-component systems; *artB* (called *pltC* in *S.* Typhi) expression is primarily regulated by SsrA-SsrB (32), while TT genes are primarily regulated by PhoP-PhoQ (30). However, both SsrA-SsrB and PhoP-PhoQ are induced under low-Mg^2+^ conditions (34, 46). As SsrB DNA binding is enhanced at low pH (47), while binding of PhoP (predicted regulator of TT genes) is not pH sensitive, our study provides additional support for a role of pH in fine-tuning transcription of TT genes. While this also suggests that regulation of *artB* and *pltB* transcription is conserved across S. enterica subspecies additional studies are needed to confirm the regulatory networks that control *artB* and *pltB* transcription in a broader range of NTS species.

At the level of toxin assembly, our experimental data suggest that the formation of heteropentameric TT binding subunits may occur, at least under some environmental conditions. The possible occurrence of PltB-ArtB interactions was confirmed by (i) two-hybrid system experiments, which showed evidence for some ArtB-PltB interactions for *S.* Javiana, and (ii) pulldown experiments, which showed consistent copurification of ArtB with PltB. However, our data also indicate that homopentamers may be the favored configuration of the binding subunit, as (i) PltB-PltB and ArtB-ArtB interactions were readily detected even in a heterologous host, while weak evidence for ArtB-PltB interactions (significant at α = 0.1) was only detected for some constructs in *S.* Javiana grown in broth, (ii) Western blot detection of PltB and ArtB after TAP suggested that low levels of ArtB and PltB are present when TTs are coimmunoprecipitated in a manner targeting the other binding units (i.e., ArtB or PltB, respectively), and (iii) competition assays demonstrate that PltB efficiently replaces ArtB in the holotoxin at neutral pH, which was in contrast to the results of the *in silico* modeling suggesting that ArtB-PltB interactions were more favorable than PltB-PltB interactions. Similar to our experimental findings, a recent study suggested that TT produced by *S.* Typhi is predominantly composed of either PltB or ArtB homopentamers, although the possibility of heteropentameric configurations could not be excluded in that study (32). As both the study described here and a previous study (32) overexpressed toxin subunits in a heterologous host, future studies examining toxin expression and assembly using untagged subunits (as tagged subunits were used in this study) under native conditions in *Salmonella* will be important for understanding the putative role of heteropentameric configurations of the TT binding subunit. Furthermore, differences in the amino acid sequences of PltA and ArtB encoded by different serovars (such as *S.* Typhi and *S.* Javiana [33]) may also need to be considered for studying the assembly of this toxin among a broader range of S. enterica serovars.

**FIG 5:**
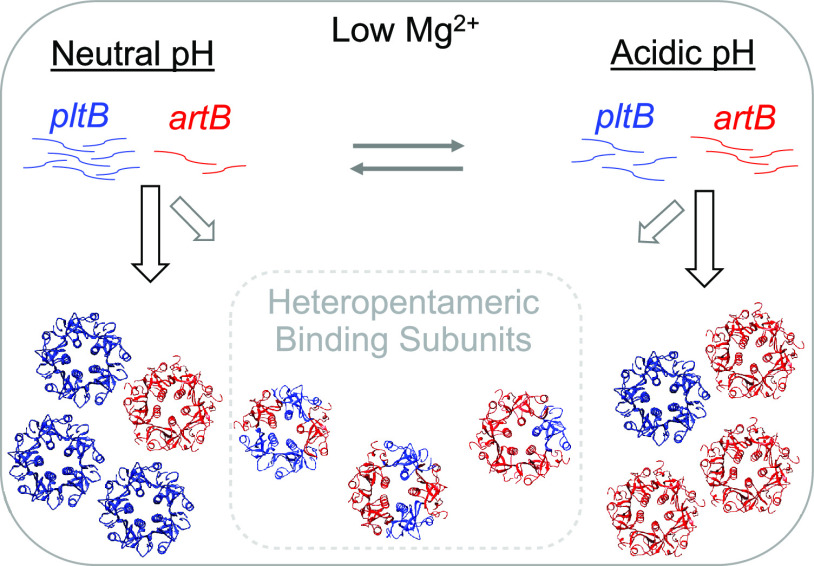
Proposed relationship between environmental cues and TT formation. Based on our data and what has been shown previously for the regulation of TT in *S.* Typhi, at neutral pH, transcription of *pltB* is induced by PhoP (30), which may lead to the preferential inclusion of PltB in the TT binding subunit. Conversely, at slightly acidic pH, SsrB induces transcription of *artB* (32) in a pH-sensitive manner (47), which may favor preferential inclusion of ArtB in the TT binding subunit. The copurification of ArtB and PltB suggests the potential formation of heteropentameric forms of the binding subunit, although a role for these heteropentameric subunits will require further investigation.

It is important to note that the experimental methods used here cannot distinguish configurations of the TT binding subunit with differing ratios of PltB and ArtB; additional structural evidence will be necessary to confirm the formation of heteropentameric configurations of the binding subunit and to assess the ratios of holotoxins with homo- and heteropentameric configurations of the binding subunit. In addition, whether the formation of ArtB-PltB heteropentameric binding subunits represents transitional forms of the TT or whether they have specific functional or virulence benefits also remains to be elucidated. One possibility is that differences in transcriptional regulation of *artB* and TT genes (*cdtB*, *pltA*, and *pltB*) resulting from changes in environmental cues (e.g., acidification of the *Salmonella*-containing vacuole [SCV]) could lead to a transitory state in which TTs with heteropentameric binding subunits represent an intermediate step that occurs as the TT transitions into a different homopentameric binding subunit (i.e., ArtB only or PltB only) (Fig. 5). Assessment of TT assembly under different conditions such as those mimicking intracellular conditions (e.g., acidified SCV) will therefore be important for procuring a more complete understanding of the potential complementary roles that ArtB and PltB play in the function of the TT. Additional experiments examining the delicate balance of transcriptional, as well as possible translational and posttranslational, regulatory mechanisms driving the formation of TT will be important for understanding the *in vivo* occurrence and any potential biological relevance of TTs with different binding subunits.

Overall, our data contribute to a growing body of evidence that suggests that ArtB plays an important and currently underappreciated role in the activity of the TT. As additional serovars are found to encode both ArtB and PltB, a more complete understanding of the TT will require a diversified approach to better assess the role that TT plays in infection with nontyphoidal serovars.

## MATERIALS AND METHODS

### Bacterial strains, plasmids, primers, and media.

Bacterial strains used in this study are listed in Table S5 in the supplemental material, vectors and recombinant constructs are listed in Table S6, and primers are listed in Table S7. Bacterial strains were routinely grown in Difco Luria-Bertani (LB) broth, pH 7, containing 5 g/liter NaCl (Becton, Dickinson [BD], Franklin Lakes, NJ). For two-hybrid system interactions, E. coli and *S.* Javiana strains were grown in M63 medium with maltose as the sole carbon source (48). N-salts minimal medium (pH 5.8 or 7) containing 8 µM MgSO_4_ (34) was used for experiments assessing transcript abundances of TT genes and *artB*. Unless otherwise indicated, ampicillin and kanamycin were used at 100 µg/ml and 50 µg/ml, respectively, in complex medium (LB broth) and 50 µg/ml and 25 µg/ml, respectively, in chemically defined media (M9 or N-salts minimal medium).

10.1128/mSphere.01255-20.7 TABLE S5 Strains used in this study. Download Table S5, PDF file, 0.1 MB. Copyright © 2021 Gaballa et al. 2021 Gaballa et al. This content is distributed under the terms of the Creative Commons Attribution 4.0 International license.

10.1128/mSphere.01255-20.8 TABLE S6 Vectors and constructs used in this study. Download Table S6, PDF file, 0.1 MB. Copyright © 2021 Gaballa et al. 2021 Gaballa et al. This content is distributed under the terms of the Creative Commons Attribution 4.0 International license.

10.1128/mSphere.01255-20.9 TABLE S7 Primers used in this study. Download Table S7, PDF file, 0.1 MB. Copyright © 2021 Gaballa et al. 2021 Gaballa et al. This content is distributed under the terms of the Creative Commons Attribution 4.0 International license.

Human intestinal epithelial cells (HIEC-6 cells; ATCC, Manassas, VA) (49) were cultured in Opti-MEM (Gibco-Invitrogen, Carlsbad, CA) supplemented with 10% (vol/vol) fetal bovine serum (FBS; Gibco-Invitrogen) and recombinant epidermal growth factor (10 ng/ml; Gibco-Invitrogen) at 37°C with 5% CO_2_. Cell supernatants were routinely tested to confirm the absence of *Mycoplasma* and *Acholeplasma* species infection using the VenorGEM *Mycoplasma* detection kit (Sigma-Aldrich, St. Louis, MO).

### Cloning and expression of toxin proteins in E. coli.

Toxin subunits were expressed in E. coli BTH101 cells (50) using (i) *pltB*-*3×-Flag*-*artB*-*c-Myc* cloned into the high-copy-number pUT18 vector or (ii) *cdtB*-*His*-*pltA*-*Flag* and *cdtB-His*-*pltA-Strep*, each cloned into the low-copy-number pKNT25 plasmid. All PCRs were performed using the high-fidelity polymerase Q5 (New England Biolabs [NEB], Ipswich, MA) and the primers listed in Table S7. All constructs were designed to contain identical ribosome binding sites (AGGAGG) 5 to 7 bases upstream of the ATG start codons. The DNA sequences of all constructs were confirmed with Sanger sequencing.

For construction of pUT18::*pltB*-*3×-Flag*-*artB*-*c-Myc*, PCR products representing (i) *pltB-3×-Flag* and (ii) *artB*-*c-Myc* were digested using (i) XbaI and KpnI and (ii) KpnI and EcoRI, respectively, and were ligated into XbaI-EcoRI-digested pUT18 using T4 DNA ligase (NEB). For construction of pKNT25::*cdtB*-*His*-*pltA*-*Flag* and pKNT25::*cdtB-His*-*pltA-Strep*, PCR products (i) *cdtB*-*His* and (ii) *pltA-Flag* or *pltA*-*Strep* were digested with (i) XbaI and KpnI and (ii) KpnI and SacI, respectively, followed by ligation into XbaI-SacI-digested pKNT25. To construct pKNT25 containing *cdtB-His* only, the *pltA-Flag* region was deleted from pKNT25::*cdtB*-*His*-*pltA*-*Flag* by digesting plasmid DNA with KpnI and SacI. The resulting 3′ overhang was removed, and the 5′ overhang was filled in with T4 DNA polymerase (NEB). Following purification, the plasmid was self-ligated with T4 DNA ligase. pKNT25::*pltA-Flag*, pUT18::*pltB-3×-Flag* and pUT18::*artB-c-Myc* were constructed in a similar fashion.

Expression of these constructs was carried out in an E. coli BTH101 cAMP-null strain (Δ*cyaA*) due to the observed toxicity of TT components in E. coli NEB5α cells (NEB). Sequence-confirmed clones were cotransformed (pUT18 and pKNT25) into E. coli BTH101 cells and were selected for ampicillin and kanamycin resistance on LB agar plus 1% (wt/vol) glucose, incubated at 37°C.

### Assessment of two-hybrid system interactions.

Interactions of CdtB, PltA, PltB, and ArtB subunits were first assessed using the bacterial adenylate cyclase two-hybrid (BACTH) system in E. coli BTH101 cells (50). The full-length polypeptides of the toxin subunits (including signal peptides) were fused to the N termini of T25 or T18 subunits in pKNT25 and pUT18, respectively (Fig. 3C). To ensure cytoplasmic CyaA activity, we also cloned the coding regions of CdtB, PltA, PltB, and ArtB lacking the signal peptide into BACTH plasmids for fusion at either the N terminus (pKNT25 and pUT18) or C terminus (pKT25 and pUT18C). Regions encoding the C-terminal interacting domains of PltA, PltB, and ArtB, as predicted from the TT structure (18, 26), were also cloned into pKNT25, pUT18, pKT25, and pUT18C plasmids (Tables S6 and S7). As 324 theoretical combinations exist, we used a high-throughput screening method. Screening was performed by cotransforming T18 and T25 vectors into E. coli BTH101 cells, selecting on LB agar plus 1% (wt/vol) glucose with ampicillin and kanamycin, and subsequently substreaking colonies onto LB agar with ampicillin and kanamycin plates containing 1 mM isopropyl-β-d-thiogalactopyranoside (IPTG) and 40 µg/ml of 5-bromo-4-chloro-3-indolyl-β-d-galactopyranoside (X-Gal) for blue-white screening. To ensure that positive interactions were not false positives arising from cAMP-independent CAP* spontaneous mutations, plasmid DNA was purified from blue colonies and retransformed into E. coli BTH101. Plasmids were isolated from clones that resulted in blue colonies after the second transformation and were analyzed by Sanger sequencing to determine the identity of the interacting domains.

### Construction of Δ*cyaA S*. Javiana.

Deletion of *cyaA* was performed to enable screening for maltose utilization resulting from an interaction of T18- and T25-fused proteins. Whole-genome sequence analysis identified a single full-length *cyaA* in the *S.* Javiana strain used in this study (FSL S5-0395). Construction of a Δ*cyaA S.* Javiana strain was performed using the λ-Red recombinase system as described previously (16). Plasmids and primers used to generate the Δ*cyaA* strain are listed in Tables S6 and S7, respectively. The in-frame deletion was confirmed by Sanger sequencing. The mutant strain was also phenotypically confirmed by a lack of growth in M63 medium containing maltose as the sole carbon source; growth was restored upon addition of 0.05 mM cAMP.

### Expression of the two-hybrid system constructs in M63 minimal medium.

Plasmids containing ArtB and PltB constructs were transformed into the Δ*cyaA S.* Javiana strain via electroporation. Transformed cells were selected on LB agar plates supplemented with ampicillin and kanamycin. Overnight cultures (12 to 14 h) were grown shaking at 30°C in LB broth with ampicillin and kanamycin. Subsequently, M63 broth containing ampicillin, kanamycin, and 0.02 mM cAMP was inoculated with an overnight culture (diluted 1:100), followed by incubation with shaking at 30°C for 24 h. The addition of 0.02 mM cAMP (a concentration that does not support the growth of negative controls) (Fig. 3D) was necessary for the basal expression of the fusion proteins in *S.* Javiana. Growth was assessed as optical density at 600 nm (OD_600_), which was measured after 24 h with a BioTek synergy plate reader (BioTek Instruments, Inc., Winooski, VT). Each growth assay was performed as three independent experiments.

### Molecular modeling of different forms of the TT binding subunit.

*In silico* modeling of TT subunits was done using the Phyre2 server (51). Construction of the TT with different ratios of PltB and ArtB was performed using the MatchMaker tool in Chimera software (52). The stability and biological relevance of homo- and heteropentamer formation were assessed by calculating free energies from individual subunit-subunit interactions within the pentamer, using the PDBe Protein Interfaces, Surfaces and Assemblies (PDBe PISA) server (53).

### Tandem affinity purification.

Beveled flasks containing 250 ml of LB broth with ampicillin and kanamycin were inoculated with overnight cultures (12 to 14 h, 1:500 dilution) of E. coli BTH101 harboring plasmids pAG37 and pAG43, which express PltB-3×-Flag ArtB-c-Myc and CdtB-His PltA-Strep, respectively. Cells were grown shaking at 200 rpm for 4.5 h at 37°C, followed by induction with IPTG and cAMP (both 1 mM final concentrations) for an additional 1.5 h. Cells were collected by centrifugation and stored at −80°C. Cells were thawed on ice, resuspended in 2 ml of NTA buffer (50 mM NaH_2_PO_4_ [pH 8.0], 0.3 M NaCl) containing 2 mg lysozyme, and incubated at 37°C for 30 min. Cell lysis was achieved with three rounds of freeze-thaw cycles (submersion in liquid nitrogen and incubation at 37°C) followed by sonication using a Branson Sonifier 250 sonicator (80% duty cycle, 7 output control) for 30 s on ice (performed twice). Cell debris was removed by centrifugation at 15,000 × *g* for 10 min. For CdtB-His purification, 50 µl of Dynabeads (Thermo Fisher Scientific; Waltham, MA) was washed twice with 500 µl of cold NTA buffer in preparation for TAP. Cell lysates were added to beads, followed by incubation overnight (12 to 14 h) at 4°C in a tube rotator. Beads were collected on a magnetic stand and were washed three times at 4°C with 500 µl of cold NTA buffer with incubation periods of 10 min. Proteins were eluted using 300 µl of NTA buffer containing 250 mM imidazole. Eluted proteins were then dialyzed against TBS buffer (50 mM Tris-Cl, 150 mM NaCl, pH 7.5) containing 10% (vol/vol) glycerol. PltB-3×-Flag was pulled down using Flag magnetic beads (EMD Millipore) according to the manufacturer’s instructions. Proteins were eluted from beads by boiling in the presence of 100 µl of 1× SDS-loading dye. PltB-3×-Flag and ArtB-c-Myc were detected in subfractions using Western blot analyses performed with rabbit anti-Flag (Sigma number [no.] F7425, diluted 1:500) and rabbit anti-c-Myc (Sigma no. SAB4301136, diluted 1:4,000) antibodies with goat anti-rabbit-horseradish peroxidase (HRP) antibodies (Thermo Fisher Scientific no. 65-6120, diluted 1:3,000). Horseradish peroxidase-conjugated secondary antibodies were detected using Clarity Western ECL substrate (Bio-Rad). Blots were visualized using the Bio-Rad ChemiDoc MP imaging system.

### Western blot detection of proteins.

Protein samples were resolved on 4% to 20% Mini-Protean TGX precast protein SDS-PAGE gels (Bio-Rad Laboratories; Hercules, CA) and blotted on polyvinylidene difluoride (PVDF) membranes using the Trans-Blot Turbo transfer system (Bio-Rad). Membranes were incubated in TBS buffer containing 0.1% (vol/vol) Tween 20 (TTBS) and 5% (wt/vol) blocking reagent (Bio-Rad) with gentle shaking at room temperature for 30 min. Primary antibodies were added as described above with mouse anti-strep (Millipore Sigma no. 71590-M, 1:1,000 dilution) and mouse anti-His antibodies (Santa Cruz Biotechnology, Dallas, TX; no. SC-53073, 1:400 dilution) diluted in TTBS with 0.5% (wt/vol) blocking reagent, followed by incubation with gentle shaking at room temperature for 12 to 14 h. Membranes were washed three times with TTBS, followed by incubation (2 h at room temperature with gentle shaking) with secondary antibodies (goat anti-rabbit-HRP [Thermo Fisher Scientific no. 65-6120, diluted 1:3,000] and donkey anti-mouse-Alexa 647 [Thermo Fisher Scientific no. A-31571, diluted 1:1,000]) diluted in TTBS with 0.5% (vol/vol) blocking reagent. Membranes were washed twice in TTBS and once in TBS before Western blot detection. Blots were visualized using the Bio-Rad ChemiDoc MP imaging system.

### Binding subunit exchange assay.

Protein expression and cell lysis of E. coli BTH101 strains (FSL G4-0035 to G4-0038) were performed as described above for TAP with cell pellets resuspended in PBS containing 10% (vol/vol) glycerol. Final total protein concentrations were determined spectrophotometrically (Nanodrop 2000c) (54, 55). The PltB-3×-Flag-containing lysate was added at various protein concentrations to 100 µg of total cell lysate containing CdtB-His, PltA-Flag, and ArtB-c-Myc for a final reaction volume of 50 µl of PBS with 10% (vol/vol) glycerol. The reaction mixture was incubated at 37°C for 30 min, followed by 2 h of incubation at 4°C to ensure equilibrium. A 200-µl aliquot of NTA buffer was added, and protein complexes were purified using Ni-nitrilotriacetic acid (NTA) beads, as described above. The assay was also performed with lysates containing CdtB-His, PltA-Flag, and PltB-3×-Flag as target and ArtB-c-Myc as the competitor. All proteins were detected by Western blotting using antibodies that recognize the corresponding protein tag (i.e., Flag, anti-His, and anti-c-Myc); three independent experiments were performed.

### Intoxication of HIEC-6 cells with crude lysates of toxin components expressed in E. coli BTH101 cells.

Fresh Opti-MEM (containing 10 ng/ml recombinant epidermal growth factor [r-EGF] and 10% [vol/vol] FBS) medium supplemented with 10 µg/ml gentamicin was added to HIEC-6 cells grown to confluence on 12-mm coverslips (Thermo Fisher Scientific) in 24-well plates (Corning Inc., Corning, NY). Lysates containing various combinations of toxin subunits were then added to HIEC-6 cells at a final concentration of 400 µg total protein per ml, and the HIEC-6 cells were subsequently incubated at 37°C with 4.5% CO_2_. Immunofluorescence (IF) detection of DDR foci and cell cycle analyses were performed at 24 ± 2 h after inoculation. Three independent experiments were performed for both cell cycle and IF detection experiments.

### IF detection of DDR proteins γH2AX and 53BP1.

IF staining for γH2AX and 53BP1 foci was performed as described previously (16, 33). The following antibodies were used: mouse anti-γH2AX (diluted 1:500, no. 05-636; Millipore Sigma, Billerica, MA), rabbit anti-53BP1 (diluted 1:500, no. NB 100–304; Novus Biologicals, Littleton, CO), donkey anti-rabbit conjugated to Alexa 555 (diluted 1:500, no. A-31572; Thermo Fisher Scientific), and donkey anti-mouse conjugated to Alexa 647 (diluted 1:200, no. A-31571; Thermo Fisher Scientific). Nuclei were stained with 4′,6-diamidino-2-phenylindole (DAPI; Thermo Fisher Scientific) for 5 min at room temperature. Microscopic observation was performed using a Zeiss 710 confocal microscope. FIJI software was used for image processing (56). Cells (at least 50 were scored per treatment) were considered to have an activated DDR if their nuclei had at least four 53BP1-positive foci and also contained γH2AX foci.

### Cell cycle analyses.

Staining with propidium iodide for cell cycle analysis determination was performed as described previously (16, 33). DNA content (for cell cycle analysis) was assessed using the FACSAria flow cytometer (BD). Gating was performed to exclude multiplets as described previously (16, 57).

### Whole-genome sequence data analyses for 235 *Salmonella* serovars.

Assemblies for the 235 S. enterica subsp. *enterica* serovars and five other S. enterica subspecies were downloaded from NCBI (see Data Set S1 for S. enterica data set). For the S. enterica data set, isolates were selected to include serovars commonly associated with human clinical disease (7) and those representing novel clades of S. enterica (29). Sequences from a convenience sample of 40 *S.* Javiana isolates, representing 28 unique SNP clusters were downloaded from the NCBI Pathogen Detection browser (https://www.ncbi.nlm.nih.gov/pathogens/isolates/#/search/) (see Table S3 for details); WGS data for *S.* Javiana FSL S5-0395 was also included. *S.* Mississippi isolate SRR1960042 was included as an outgroup for phylogenetic analyses (Table S3). For these data, Illumina adapters from sequence reads were trimmed, and low-quality bases were removed using Trimmomatic 0.33 with default settings (58). Determination of the quality of trimmed reads was performed using FastQC v0.11.7 (59). *De novo* assembly of all genomes was performed using SPAdes 3.6.0 (60). To assess the qualities of draft genomes, QUAST 3.2 (61) was used, followed by BBmap 35.49 (62) and SAMtools 1.3.1 (63) to calculate average coverage. Serotypes were confirmed using SISTR (64). kSNP3 was used to identify core SNPs in (i) the S. enterica data set and (ii) 40 *S.* Javiana genomes and strain FSL S5-0395; a *k*-mer size of 19 was used for both data sets, as determined using kSNP3’s Kchooser function (65). Phylogenetic trees were constructed with RAxML (66) using a general time-reversible model with gamma-distributed sites constructed from either 100 (S. enterica data set) or 1,000 (*S.* Javiana data set) bootstrap repetitions. iTOL v4 was used for editing of phylogenetic trees (67, 68).

10.1128/mSphere.01255-20.10 DATA SET S1 Predicted serotype data for whole-genome assemblies used in analyses included in Fig. 1. Download Data Set S1, XLSX file, 0.1 MB. Copyright © 2021 Gaballa et al. 2021 Gaballa et al. This content is distributed under the terms of the Creative Commons Attribution 4.0 International license.

### Detection of TT genes and *artAB*.

The presence of *artA*, *artB*, and TT genes was queried for all isolates using nucleotide BLAST (blastn) version 2.3.0 with default settings to query *artA*, *artB*, *pltA*, *cdtB*, and *pltB* sequences from *S.* Javiana strain CFSAN001992 (69); an E value of 1e−20 was used for querying *S.* Javiana genomes, and an E value of 1e−10 was used for querying the 235 serovars. In addition, closed genomes were downloaded to visually assess the location of TT genes and *artB* in serovars representing different phylogenetic clades (see Table S2 for assembly and strain information). Genomes were selected if they (i) represented commonly used strains from serovars associated previously with outbreaks of human clinical illness (e.g., CT18 for *S.* Typhi, AKU_12601 for *S.* Paratyphi A, CFSAN001992 for *S.* Javiana, and ATCC BAA-1673 for *S.* Poona) or (ii) were among the few closed genomes available for serovars that were positive for both TT and *artB* (serovars *S.* Goldcoast [clade A1] and *S.* Milwaukee [clade A2]); serovar *S.* Indiana was selected as an additional serovar for assessing the genomic location of toxin genes in section Typhi serovars, as *S.* Paratyphi A and *S.* Typhi represented a unique phylogenetic clade in our core genome analysis (Fig. 1A). In addition, to confirm the absence of *artB* in select *S.* Javiana assemblies, mapping of raw reads to *artB* from CFSAN001992 was performed using BWA version 0.7.17.

### RT-qPCR quantification of *artB* and *pltB* transcript levels.

Overnight cultures (16 to 18 h) of FSL S5-0395 grown in LB broth were subcultured 1:1,000 into LB or N-salts minimal medium (either pH 7 or pH 5.8), and subcultured samples were grown at 37°C with shaking at 200 rpm until cells reached mid-exponential phase (3 h for LB broth, 5 h for N-salts minimal medium). RNA was stabilized with RNA protect (Qiagen) and was collected using the RNeasy kit (Qiagen). DNA was depleted with Ambion DNase I (Life Technologies), and DNA depletion was confirmed with quantitative PCR (qPCR); a cycle threshold (*C_T_*) of 34 for *rpoB* in DNase-treated RNA samples was used for judging successful DNA depletion. cDNA libraries were prepared with the Superscript reverse transcription kit (Thermo Fisher), according to manufacturer’s instructions. qPCR was performed with SYBR green 2× master mix (Applied Biosystems) in a reaction mixture containing 0.4 μM each primer (see Table S7) and 1 µl of cDNA (approximately 15 ng of cDNA) as the template. Fold expression was calculated by raising the ΔΔ*C_T_* to the power of the efficiency calculated for each primer pair (70). Results are the averages from three independent experiments, each performed in technical duplicates.

### Statistical analyses.

Statistical differences were assessed using R studio version 3.4.2. using packages lme4 (71) 1.1-14, emmeans version 1.3.3 (72), lmerTest version 2.0-33 (73), and multicomp version 1.4-8 (74) for Dunnett’s test for multiple comparisons adjustment.

### Data availability.

Scripts and data sets are available online at https://github.com/ram524/2019_ArtB.
