# Supplementary material for: The Majority of Typhoid Toxin-Positive Salmonella Serovars Encode ArtB, an Alternate Binding Subunit
Source: mSphere. 2021 Jan 6;6(1):e01255-20. doi: 10.1128/mSphere.01255-20 (PMC7845599; doi:10.1128/mSphere.01255-20)
Supplement: TABLE S1 [file mSphere.01255-20-st001.pdf]

Subspecies	subsp. *enterica* clade[a]	Serovar	Assembly	***pltA* length (% identity with *S.* Javiana *pltA*)[b]**	***pltB* length (% identity with *S.* Javiana *pltB*)[c]**
arizonae	-	IIIa 41:z_4,z_{23}:-	GCA_000018625	732 nt. (81%)	456 nt. (67%)
diarizonae	-	IIIb 50:r:z	GCF_002107575	732 nt. (82%)	453 nt. (68%)
enterica	A2	Inverness	GCA_000487155	732 nt. (81%)	426 nt. (65%)
enterica	B	Tanger	GCA_007009285	738 nt. (79%)	423 nt. (67%)
enterica	D	Brazzaville	GCA_007644865	735 nt. (79%)	414 nt. (66%)
enterica	D	Kalina	GCA_007414785	735 nt. (79%)	414 nt. (66%)
enterica	D	Westminster	GCA_009249885	735 nt. (79%)	414 nt. (66%)
enterica	Section Typhi	Mississippi	GCA_008588385	732 nt. (82%)	414 nt. (67%)

[a]clade assigned based on phylogenetic assessment from core SNP analysis.

[b]comparison of *pltA* in given genome with *pltA* from *S.* Javiana CFSAN001992, which has a nucleotide length of 729 nt.; Inverness GCA_000487155 encodes two different TT islets that share 100% identity with each other, therefore the results presented here are representative of both loci.

[c]comparison of *pltB* in given genome with *pltB* from *S.* Javiana CFSAN001992, which has a nucleotide length of 414 nt.; Inverness GCA_000487155 encodes two different TT islets that share 100% identity with each other, therefore the results presented here are representative of both loci.
