# Supplementary material for: The Majority of Typhoid Toxin-Positive Salmonella Serovars Encode ArtB, an Alternate Binding Subunit
Source: mSphere. 2021 Jan 6;6(1):e01255-20. doi: 10.1128/mSphere.01255-20 (PMC7845599; doi:10.1128/mSphere.01255-20)
Supplement: TABLE S2 [file mSphere.01255-20-st002.pdf]

Clade[a]	Serovar	Strain	Assembly	Approx. *artB* islet location	Number of ORFs[b]	*artB* islet % GC[c]	Genome % GC
A1	Goldcoast	R18.0877	CP037960	2.16 – 2.17 Mb	5	41.7%	52.3%
A2	Milwaukee	SA19950795	CP030175	2.47 – 2.48 Mb	4	44.7%	52.2%
B	Javiana	CFSAN001992	CP004027	0.56 – 0.57 Mb	5	41.6%	52.2%
B	Poona	ATCC BAA-1673	CP019189	2.17 – 2.18 Mb	5	41.2%	52.2%
Typhi	Indiana	SI102	CP050771	2.24 – 2.25 Mb	5	41.9%	52.1%
Typhi	Paratyphi A	AKU_12601	AFM200053	1.24 – 1.25 Mb	5	41.9%	52.2%
Typhi	Typhi	CT18	AL513382	1.31 – 1.32 Mb	7	42.6%	52.1%

[a]Clade assigned based on phylogenetic assessment from core SNP analysis.

[b]The number of open reading frames (ORFs) assigned by NCBI annotation that were at least 100 nucleotides in length.

[c]*artB* islet includes all nucleotides between *sapB* and *sapC.*
