# Supplementary material for: The Majority of Typhoid Toxin-Positive Salmonella Serovars Encode ArtB, an Alternate Binding Subunit
Source: mSphere. 2021 Jan 6;6(1):e01255-20. doi: 10.1128/mSphere.01255-20 (PMC7845599; doi:10.1128/mSphere.01255-20)
Supplement: TABLE S3 [file mSphere.01255-20-st003.pdf]

Serovar[a]	Run #[b]	Source	Country[c]	SNP Cluster[d]	Reason for Inclusion
Javiana	SRR11604455	Human clinical	USA	Not listed	Used for experiments
Javiana	SRR7081896	Human clinical (stool)	USA	Not listed	Clinical source
Javiana	SRR7063447	Human clinical (blood)	USA	PDS000026970	Clinical source
Javiana	SRR7063441	Human clinical (stool)	USA	PDS000026970	Clinical source
Javiana	SRR7063436	Human clinical (stool)	USA	PDS000026970	Clinical source
Javiana	SRR5816109	Animal (swine)	USA	PDS000026915	Unique source
Javiana	SRR6311987	Animal (canine)	USA	PDS000036032	Unique source
Javiana	SRR5598907	Environmental (frog legs)	USA	PDS000027056	Unique source
Javiana	SRR2585797	Environmental (toad)	Mexico	PDS000027026	Unique source
Javiana	SRR2968972	Environmental (creek water)	USA	PDS000026970	Unique source
Javiana	SRR4124058	Human clinical (stool)	USA	Not listed	Clinical source
Javiana	SRR6480222	Human clinical (stool)	USA	PDS000002099	Clinical source
Javiana	SRR6396486	Human clinical (stool)	USA	PDS000026970	Clinical source
Javiana	SRR6321401	Human clinical (stool)	USA	PDS000002101	Unique SNP cluster
Javiana	SRR5724832	Human clinical (stool)	USA	PDS000002099	Clinical source
Javiana	SRR5676348	Human clinical (stool)	USA	PDS000026970	Clinical source
Javiana	SRR5605812	Human clinical (urine)	USA	PDS000026970	Clinical source
Javiana	SRR1509381	Human clinical (stool)	USA	PDS000031655	Unique SNP cluster
Javiana	SRR1561167	Food (lobster)	Indonesia	Not listed	Unique location
Javiana	SRR1962283	Human clinical	UK	Not listed	Clinical source
Javiana	SRR1965602	Human clinical	UK	PDS000031042	Unique SNP cluster
Javiana	SRR3092111	Food (peppers)	Mexico	Not listed	Unique source
Javiana	SRR3137186	Human clinical (stool)	USA	PDS000026970	Clinical source
Javiana	SRR5152112	Human clinical (stool)	USA	PDS000030196	Unique SNP cluster
Javiana	SRR5160225	Human clinical (stool)	USA	PDS000027927	Unique SNP cluster
Javiana	SRR5289505	Food (candy)	Pakistan	PDS000001767	Unique location
Javiana	SRR5429767	Human clinical (stool)	USA	PDS000002100	Unique SNP cluster
Javiana	SRR5508324	Human clinical (stool)	USA	PDS000002089	Unique SNP cluster
Javiana	SRR5583207	Human clinical	UK	PDS000028979	Unique SNP cluster
Javiana	SRR5725238	Human clinical (stool)	USA	PDS000026937	Unique SNP cluster
Javiana	SRR7346572	Human clinical (stool)	USA	PDS000026970	Unique SNP cluster
Javiana	SRR6791758	Human clinical (stool)	USA	PDS000027037	Unique SNP cluster
Javiana	SRR5217754	Human clinical (stool)	USA	PDS000026970	Clinical source
Javiana	SRR5217761	Human clinical (stool)	USA	PDS000026982	Unique SNP cluster
Javiana	SRR6425474	Human clinical (stool)	USA	PDS000027004	Unique SNP cluster
Javiana	SRR5209739	Human clinical (blood)	USA	PDS000026915	Unique SNP cluster
Javiana	SRR5023225	Human clinical (stool)	USA	PDS000026926	Unique SNP cluster
Javiana	SRR6760627	Human clinical (stool)	USA	PDS000026948	Unique SNP cluster
Javiana	SRR2601212	Human clinical (stool)	USA	PDS000027935	Unique SNP cluster
Javiana	SRR1754882	Human clinical (stool)	USA	PDS000031372	Unique SNP cluster
Javiana	SRR8082595	Food (kratom)	USA	PDS000027723	Unique source
Mississippi	SRR1960042	Human clinical	UK	PDS000006090	Outgroup

[a]Serovar confirmed with SISTR
[b]SRR run ID assigned by NCBI
[c]Country of origin
[d]SNP cluster assigned by NCBI’s Pathogen Browser website
