# Supplementary figures and images for: The Majority of Typhoid Toxin-Positive Salmonella Serovars Encode ArtB, an Alternate Binding Subunit
Source: mSphere. 2021 Jan 6;6(1):e01255-20. doi: 10.1128/mSphere.01255-20 (PMC7845599; doi:10.1128/mSphere.01255-20)

**A****5 ArtB**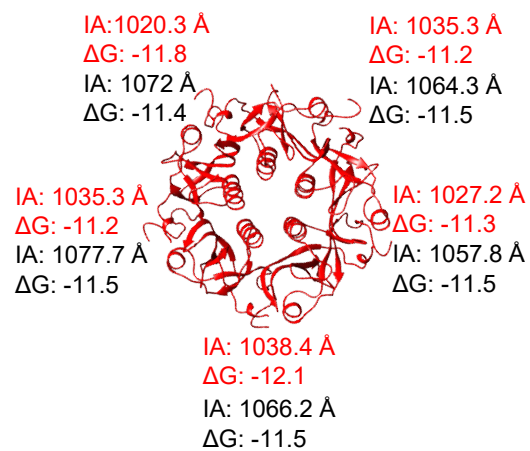

**S. Typhimurium DT104**  
**S. Javiana CFSAN001992**

**B**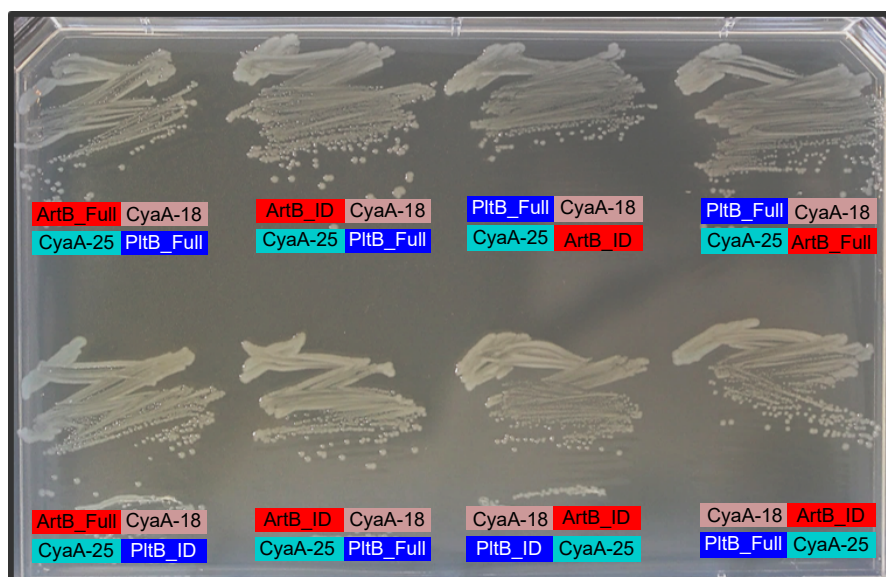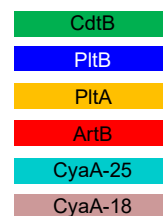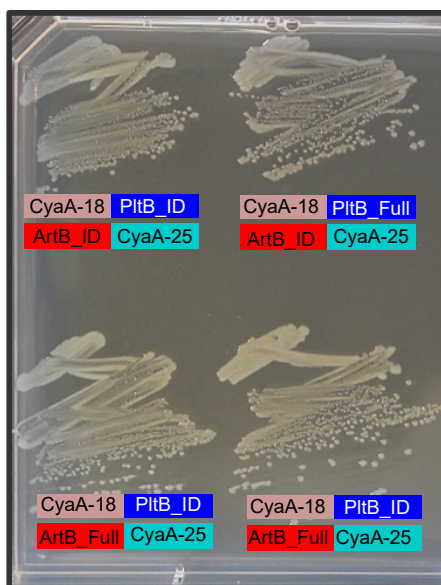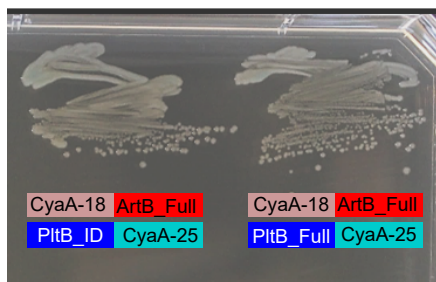

Supplement: FIG S1 [file mSphere.01255-20-sf001.pdf]

**A****Strain**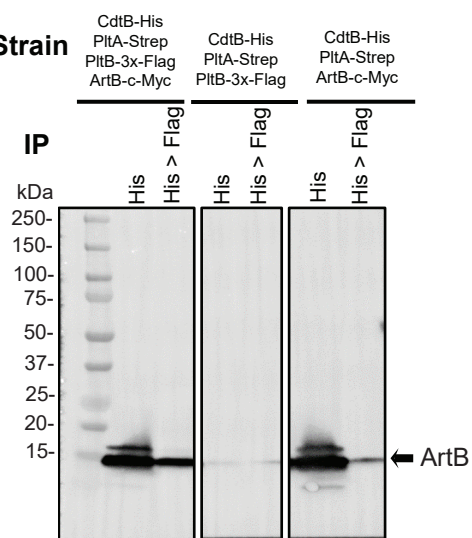**B**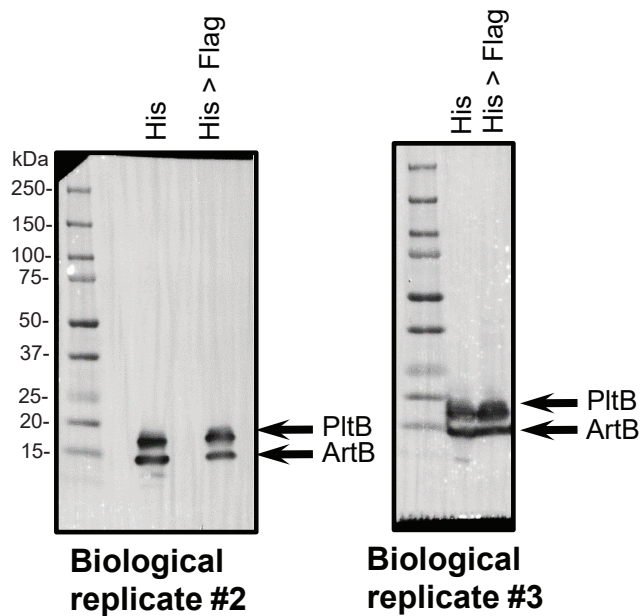**C**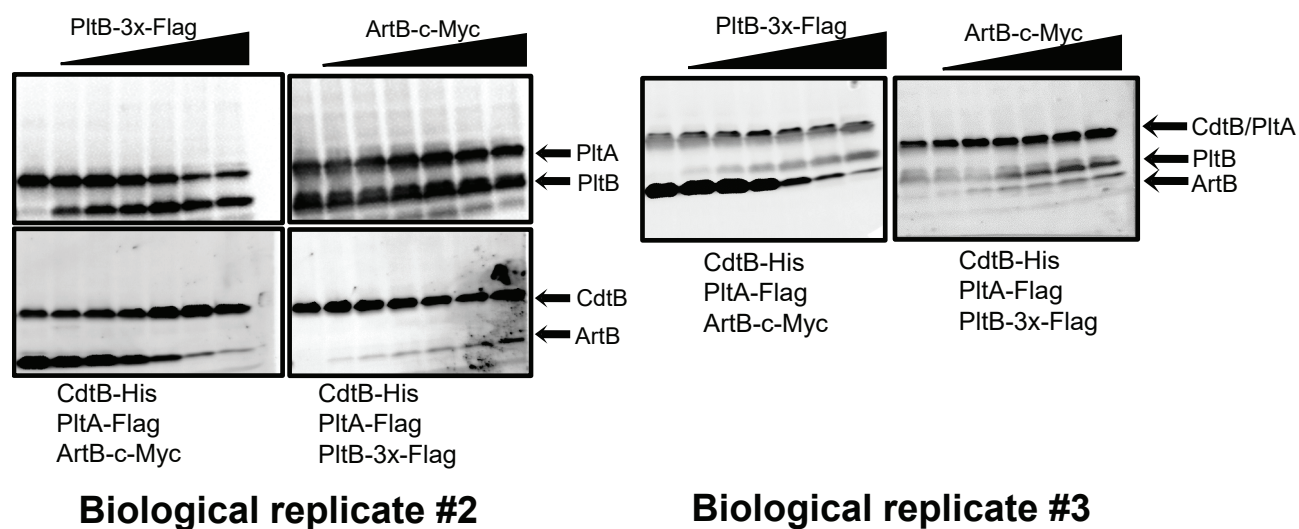

Supplement: FIG S2 [file mSphere.01255-20-sf002.pdf]
