# Supplementary material for: The Majority of Typhoid Toxin-Positive Salmonella Serovars Encode ArtB, an Alternate Binding Subunit
Source: mSphere. 2021 Jan 6;6(1):e01255-20. doi: 10.1128/mSphere.01255-20 (PMC7845599; doi:10.1128/mSphere.01255-20)
Supplement: TABLE S4 [file mSphere.01255-20-st004.pdf]

Plasmid combinations[a]	Estimate	Standard Error	t value	p value	p value adjust[b]
$ArtB_{T25N}$ + $ctPltB_{T18C}$	-0.030360	0.043426	-0.699	0.48897	0.9995
$ArtB_{T25N}$ + $PltB_{T18C}$	-0.003575	0.043426	-0.082	0.93485	1.0000
$ArtB_{T18}$ + $ArtB_{T25}$	0.317006	0.043426	7.300	1.33e-08	<0.001
$ArtB_{T18}$ + $ctPltB_{T25}$	0.011286	0.043426	0.260	0.79643	1.0000
$ArtB_{T18C}$ + $ctPltB_{T25}$	-0.011878	0.043426	-0.274	0.78602	1.0000
$ArtB_{T18C}$ + $PltB_{T25}$	-0.022905	0.043426	-0.527	0.60112	1.0000
$ctArtB_{T25}$ + $PltB_{T18C}$	0.014355	0.043426	0.331	0.74289	1.0000
$ctArtB_{T25}$ + $PltB_{T18}$	0.112489	0.043426	2.590	0.01376	0.1353
$ctArtB_{T18}$ + $PltB_{T25}$	0.004132	0.043426	0.095	0.92472	1.0000
$ctArtB_{T18C}$ + $ArtB_{NT25}$	0.060729	0.043426	1.398	0.17054	0.8166
$ctArtB_{T18C}$ + $ctPltB_{NT25}$	-0.038878	0.043426	-0.895	0.37659	0.9924
$ctArtB_{T18C}$ + $PltB_{NT25}$	0.037090	0.043426	0.854	0.39870	0.9952
$ctPltB_{T18}$ + $ctArtB_{T25}$	0.038253	0.043426	0.881	0.38423	0.9935
$ctPltB_{T18}$ + $ctPltB_{T25}$	1.155483	0.043426	26.608	<2e-16	<0.001
$PltB_{T25}$ + $ArtB_{T18}$	0.119244	0.043426	2.746	0.00936	0.0973
$PltB_{T25}$ + $ctArtB_{T18}$	-0.027102	0.043426	-0.624	0.53651	0.9999
$PltB_{T18}$ + $PltB_{T25}$	1.274816	0.043426	29.356	<2e-16	<0.001
Positive control	1.333219	0.043426	30.701	<2e-16	<0.001

[a]subunits are abbreviated as T25 and T18 for the CyaA fusion where “T25” indicates C-terminus fusion, “T25N” N-terminus fusion, “T18” N-terminus fusion and “T18C” C-terminus fusion to the subunit; “ct” reflects that only the C-terminus of the subunit was included in the construct.
[b]p-value after multiple comparison adjustment with Dunnett contrast.
