# Supplementary material for: The Majority of Typhoid Toxin-Positive Salmonella Serovars Encode ArtB, an Alternate Binding Subunit
Source: mSphere. 2021 Jan 6;6(1):e01255-20. doi: 10.1128/mSphere.01255-20 (PMC7845599; doi:10.1128/mSphere.01255-20)
Supplement: TABLE S5 [file mSphere.01255-20-st005.pdf]

Strain	Relevant genotype/markers	Source
BTH101	F^-, *cya-99, araD139, galE15, galK16, rpsL1 (Str^r), hsdR2, mcrA1, mcrB1*	(1)
NEB5α	*fhuA2 Δ(argF-lacZ)U169 phoA glnV44 Φ80 Δ(lacZ)M15 gyrA96 recA1 relA1 endA1 thi-1 hsdR17*	New England Biolabs
FSL G4-0003	BTH101 containing pKT25 and pUT18C	This study
FSL G4-0004	BTH101 containing pKT25-zip and pUT18C-zip	This study
FSL G4-0005	BTH101 containing pAG37 and pAG40	This study
FSL G4-0006	BTH101 containing pAG37 and pAG43	This study
FSL G4-0007	BTH101 containing pAG40	This study
FSL G4-0008	BTH101 containing pAG38	This study
FSL G4-0009	BTH101 containing pAG39	This study
FSL G4-0010	BTH101 containing pAG41	This study
FSL G4-0011	BTH101 containing pAG42	This study
FSL G4-0014	BTH101 containing pAG23 and pAG25	This study
FSL G4-0015	BTH101 containing pAG19 and pAG21	This study
FSL G4-0016	BTH101 containing pAG29 and pAG31	This study
FSL G4-0017	BTH101 containing pAG34 and pAG36	This study
FSL G4-0018	BTH101 containing pAG25 and pAG29	This study
FSL G4-0019	BTH101 containing pAG25 and pAG33	This study
FSL G4-0020	BTH101 containing pAG19 and pAG31	This study
FSL G4-0021	BTH101 containing pAG19 and pAG35	This study
FSL G4-0022	BTH101 containing pAG21 and pAG33	This study
FSL G4-0023	BTH101 containing pAG21 and pAG29	This study
FSL G4-0024	BTH101 containing pAG23 and pAG31	This study
FSL G4-0025	BTH101 containing pAG19 and pAG35	This study
FSL G4-0026	BTH101 containing pAG24 and pAG36	This study
FSL G4-0027	BTH101 containing pAG20 and pAG36	This study
FSL G4-0028	BTH101 containing pAG25 and pAG34	This study
FSL G4-0029	BTH101 containing pAG22 and pAG34	This study
FSL G4-0030	BTH101 containing pAG24 and pAG32	This study
FSL G4-0031	BTH101 containing pAG26 and pAG32	This study
FSL G4-0032	BTH101 containing pAG26 and pAG30	This study
FSL G4-0033	BTH101 containing pAG22 and pAG30	This study
FSL G4-0034	BTH101 containing pAG5 and pAG9	This study
FSL G4-0035	BTH101 containing pAG40 and pAG38	This study
FSL G4-0036	BTH101 containing pAG40 and pAG39	This study

FSL S5-0395	*S.* Javiana wild-type	(2)
FSL H9-0054	*S.* Javiana Δ*cyaA*	This study

References

1. Karimova G, Ullmann A, Ladant D. 2001. Protein-protein interaction between *Bacillus stearothermophilus* tyrosyl-tRNA synthetase subdomains revealed by a bacterial two-hybrid system. J Mol Microbiol Biotechnol 3:73-82.
2. Miller RA, Wiedmann M. 2016. The cytolethal distending toxin produced by nontyphoidal *Salmonella* serotypes Javiana, Montevideo, Oranienburg, and Mississippi induces DNA damage in a manner similar to that of serotype Typhi. MBio 7.
