# Supplementary material for: The Majority of Typhoid Toxin-Positive Salmonella Serovars Encode ArtB, an Alternate Binding Subunit
Source: mSphere. 2021 Jan 6;6(1):e01255-20. doi: 10.1128/mSphere.01255-20 (PMC7845599; doi:10.1128/mSphere.01255-20)
Supplement: TABLE S6 [file mSphere.01255-20-st006.pdf]

Plasmid	Relevant genotype/markers	Source
pKT25	Kan^R, C-terminus fusion of adenylate cyclase T25 domain	(1, 2)
pKNT25	Kan^R, N-terminus fusion of adenylate cyclase T25 domain	(1, 2)
pUT18C	Amp^R, C-terminus fusion of adenylate cyclase T18 domain	(1, 2)
pUT18	Amp^R, N-terminus fusion of adenylate cyclase T18 domain	(1, 2)
pKT25-zip	Kan^R, positive control for adenylate cyclase two-hybrid system	(1, 2)
pUT18C-zip	Amp^R, positive control for adenylate cyclase two-hybrid system	(1, 2)
pAG1	pKNT25 containing full-length *cdtB* fused to adenylate cyclase T25 domain, Kan^R	This study
pAG2	pUT18 containing full-length *cdtB* fused to adenylate cyclase T18 domain, Amp^R	This study
pAG3	pKT25 containing *cdtB* lacking signal peptide coding region fused to adenylate cyclase T25 domain, Kan^R	This study
pAG4	pKNT25 containing *cdtB* lacking signal peptide coding region fused to adenylate cyclase T25 domain, Kan^R	This study
pAG5	pUT18 containing *cdtB* lacking signal peptide coding region fused to adenylate cyclase T18 domain, Amp^R	This study
pAG6	pUT18C containing *cdtB* lacking signal peptide coding region fused to adenylate cyclase T18 domain, Amp^R	This study
pAG7	pKNT25 containing full-length *pltA* fused to adenylate cyclase T25 domain, Kan^R	This study
pAG8	pUT18 containing full-length *pltA* fused to adenylate cyclase T18 domain, Amp^R	This study
pAG9	pKT25 containing *pltA* lacking signal peptide coding region fused to adenylate cyclase T25 domain, Kan^R	This study
pAG10	pKNT25 containing *pltA* lacking signal peptide coding region fused to adenylate cyclase T25 domain, Kan^R	This study
pAG11	pUT18 containing *pltA* lacking signal peptide coding region fused to adenylate cyclase T18 domain, Amp^R	This study
pAG12	pUT18C containing *pltA* lacking signal peptide coding region fused to adenylate cyclase T18 domain, Amp^R	This study
pAG13	pKT25 containing c-terminus region of *pltA* fused to adenylate cyclase T25 domain, Kan^R	This study
pAG14	pKNT25 containing c-terminus region of *pltA* fused to adenylate cyclase T25 domain, Kan^R	This study
pAG15	pUT18 containing c-terminus region of *pltA* fused to adenylate cyclase T18 domain, Amp^R	This study
pAG16	pUT18C containing c-terminus region of *pltA* fused to adenylate cyclase T18 domain, Amp^R	This study
pAG17	pKNT25 containing full-length *pltB* fused to adenylate cyclase T25 domain, Kan^R	This study
pAG18	pUT18 containing full-length *pltB* fused to adenylate cyclase T18 domain, Amp^R	This study
pAG19	pKT25 containing *pltB* lacking signal peptide coding region fused to adenylate cyclase T25 domain, Kan^R	This study
pAG20	pKNT25 containing *pltB* lacking signal peptide coding region fused to adenylate cyclase T25 domain, Kan^R	This study
pAG21	pUT18 containing *pltB* lacking signal peptide coding region fused to adenylate cyclase T18 domain, Amp^R	This study
pAG22	pUT18C containing *pltB* lacking signal peptide coding region fused to adenylate cyclase T18 domain, Amp^R	This study
pAG23	pKT25 containing c-terminus region of *pltB* fused to adenylate cyclase T25 domain, Kan^R	This study
pAG24	pKNT25 containing c-terminus region of *pltB* fused to adenylate cyclase T25 domain, Kan^R	This study
pAG25	pUT18 containing c-terminus region of *pltB* fused to adenylate cyclase T18 domain, Amp^R	This study
pAG26	pUT18C containing c-terminus region of *pltB* fused to adenylate cyclase T18 domain, Amp^R	This study
pAG27	pKNT25 containing full-length *artB* fused to adenylate cyclase T25 domain, Kan^R	This study
pAG28	pUT18 containing full-length *artB* fused to adenylate cyclase T18 domain, Amp^R	This study
pAG29	pKT25 containing *artB* lacking signal peptide coding region fused to adenylate cyclase T25 domain, Kan^R	This study
pAG30	pKNT25 containing *artB* lacking signal peptide coding region fused to adenylate cyclase T25 domain, Kan^R	This study
pAG31	pUT18 containing *artB* lacking signal peptide coding region fused to adenylate cyclase T18 domain, Amp^R	This study
pAG32	pUT18C containing *artB* lacking signal peptide coding region fused to adenylate cyclase T18 domain, Amp^R	This study
pAG33	pKT25 containing c-terminus region of *artB* fused to adenylate cyclase T25 domain, Kan^R	This study

pAG34	pKNT25 containing c-terminus region of *artB* fused to adenylate cyclase T25 domain, Kan^R	This study
pAG35	pUT18 containing c-terminus region of *artB* fused to adenylate cyclase T18 domain, Amp^R	This study
pAG36	pUT18C containing c-terminus region of *artB* fused to adenylate cyclase T18 domain, Amp^R	This study
pAG37	pUT18 containing *pltB-3x-Flag artB-c-Myc*	This study
pAG38	pUT18 containing *pltB-3x-Flag*	This study
pAG39	pUT18 containing *artB-c-Myc*	This study
pAG40	pKNT25 containing *cdtB-His pltA-Flag*	This study
pAG41	pKNT25 containing *cdtB-His*	This study
pAG42	pKNT25 containing *pltA-Flag*	This study
pAG43	pKNT25 containing *cdtB-His pltA-Strep*	This study
pKD46	λ-Red recombinase plasmid	(3)
pKD4	Template plasmid	(3)
pCP20	Flippase plasmid	(3)

References

1. Karimova G, Pidoux J, Ullmann A, Ladant D. 1998. A bacterial two-hybrid system based on a reconstituted signal transduction pathway. Proc Natl Acad Sci U S A 95:5752-6.
2. Karimova G, Ullmann A, Ladant D. 2001. Protein-protein interaction between *Bacillus stearothermophilus* tyrosyl-tRNA synthetase subdomains revealed by a bacterial two-hybrid system. J Mol Microbiol Biotechnol 3:73-82.
3. Datsenko KA, Wanner BL. 2000. One-step inactivation of chromosomal genes in *Escherichia coli* K-12 using PCR products. Proc Natl Acad Sci U S A 97:6640-5.
