# Supplementary material for: The Majority of Typhoid Toxin-Positive Salmonella Serovars Encode ArtB, an Alternate Binding Subunit
Source: mSphere. 2021 Jan 6;6(1):e01255-20. doi: 10.1128/mSphere.01255-20 (PMC7845599; doi:10.1128/mSphere.01255-20)
Supplement: TABLE S7 [file mSphere.01255-20-st007.pdf]

Primer name	Sequence (5' to 3')	Usage
AG67-21-cdtB-BACTH-R	CTTAGGTACCCGACAGCTTCGTGCCAAAAAGGCT	Clone full-length *cdtB* in BACTH vectors
AG67-41-rbs-cdtB-F	CGCTCTAGAATAGCGGGAGAGTAGATATCATGAA	Clone full-length *cdtB* in BACTH vectors
AG67-20-cdtB-BACTH-F	TCGACTCTAGAGAATATCAGTGACTACAAAGTTATG	Clone *cdtB* without signal sequence in BACTH vectors
AG67-21-cdtB-BACTH-R	CTTAGGTACCCGACAGCTTCGTGCCAAAAAGGCT	Clone *cdtB* without signal sequence in BACTH vectors
AG67-13-PltA-BACTH-R	CTTAGGTACCCGTTTAGAAAGTATAAGTTCTATTACA	Clone full-length *pltA* in BACTH vectors
AG67-37-rbs-pltA-F	CGCTCTAGAATAGAGGAGGAAGAAATAATGAAAAAGTTAATA	Clone full-length *pltA* in BACTH vectors
AG67-12-pltA-BACTH-F	TCGACTCTAGAGGTAGATTTTGTGTATCGTGTGGA	Clone *pltA* without signal sequence in BACTH vectors
AG67-13-PltA-BACTH-R	CTTAGGTACCCGTTTAGAAAGTATAAGTTCTATTACA	Clone *pltA* without signal sequence in BACTH vectors
AG67-39-rbs-artB-F	CGCTCTAGAATAGAGGAGGTGGATTATGAAAAAGAAATTAAAG	Clone *pltA* C-terminus region in BACTH vectors
AG67-13-PltA-BACTH-R	CTTAGGTACCCGTTTAGAAAGTATAAGTTCTATTACA	Clone *pltA* C-terminus region in BACTH vectors
AG67-36-stop-pltB-F	GCGTCTAGAATAATGTATATAAATAAGTTTGTGCCT	Clone full-length *pltB* in BACTH vectors
AG67-15-pltB-BACTH-R	CTTAGGTACCCGCTTGGGTCCAAAGCATTGTGT	Clone full-length *pltB* in BACTH vectors
AG67-14-PltB-BACTH-F	TCGACTCTAGAGGAGTGGACAGGAGATAAAACGA	Clone *pltB* without signal sequence in BACTH vectors
AG67-15-pltB-BACTH-R	CTTAGGTACCCGCTTGGGTCCAAAGCATTGTGT	Clone *pltB* without signal sequence in BACTH vectors
AG67-68-pltB-Ct-F	TCGACTCTAGAGAGCATATGGGCACCCTCCT	Clone *pltB* C-terminus region in BACTH vectors
AG67-15-pltB-BACTH-R	CTTAGGTACCCGCTTGGGTCCAAAGCATTGTGT	Clone *pltB* C-terminus region in BACTH vectors
AG67-39-rbs-artB-F	CGCTCTAGAATAGAGGAGGTGGATTATGAAAAAGAAATTAAAG	Clone full-length *artB* in BACTH vectors

AG67-19-ArtB-BACTH-R	CTTAGGTACCCGATTTGTCAACATAGGCCCCATA	Clone full-length *artB* in BACTH vectors
AG67-18-ArtB-BACTH-F	TCGACTCTAGAGGCTATGGCTGATTATGATACGTA	Clone *artB* without signal sequence in BACTH vectors
AG67-19-ArtB-BACTH-R	CTTAGGTACCCGATTTGTCAACATAGGCCCCATA	Clone *artB* without signal sequence in BACTH vectors
AG67-67-ArtB-Ct-F	TCGACTCTAGAGGGGAATCATAAACAGGGGTTTG	Clone *artB* C-terminus region in BACTH vectors
AG67-19-ArtB-BACTH-R	CTTAGGTACCCGATTTGTCAACATAGGCCCCATA	Clone *artB* C-terminus region in BACTH vectors
AG67-35-rbs-pltB-F	CGCTCTAGAATAGATTTTAAATGTACAGGAGAGTA	Construction of *pltB-3x-Flag*
AG67-48-pltB-3xFLAG-R1	ATCTTTATAATCACCATCATGATCTTTATAATCTGGACGCTTGGGTCCAAAGCATTGTG	Construction of *pltB-3x-Flag*
AG67-49-pltB-3xFLAG-R2	ATCATCATCTTTATAATCAATATCATGATCTTTATAATCACCATCATGATCT	Construction of *pltB-3x-Flag*
AG67-50-pltB-3xFLAG-R3	CGCGGTACCTTATTTATCATCATCATCTTTATAATCAATATCATGATCTT	Construction of *pltB-3x-Flag*
AG67-51-artB-cmyc-F	CGCGGTACCATAGAGGAGGTGGATTATGAAAAAGAAATTAAAG	Construction of *artB-c-Myc*
AG67-52-artB-cmyc-R1	TAATCAGTTTCTGTTCTGGACGATTTGTCAACATAGGCCCCAT	Construction of *artB-c-Myc*
AG67-53-artB-cmyc-R2	CGCGAATTCTTACAGATCTTCTTCGCTAATCAGTTTCTGTTCTGGACGA	Construction of *artB-c-Myc*
AG67-41-rbs-cdtB-F	CGCTCTAGAATAGCGGGAGAGTAGATATCATGAA	Construction of *cdtB-His*
AG67-54-cdtB-His-R1	GATGATGATGATGATGTGGACGACAGCTTCGTGCCAAAAAGG	Construction of *cdtB-His*
AG67-55-cdtB-His-R2	TATGGTACCTTAATGATGATGATGATGATGATGATGATGATGTGGAC	Construction of *cdtB-His*
AG67-56-pltA-FLAG-F	CGCGGTACCATAGAGGAGGAAGAAATAATGAAAAAGTTAATA	Construction of *pltA-Flag*
AG67-57-pltA-FLAG-R1	TCTTTATAATCTGGACGTTTAGAAAGTATAAGTTCTATTACA	Construction of *pltA-Flag*
AG67-58-pltA-FLAG-R2	CGCGAGCTCTTATTTATCATCATCATCTTTATAATCTGGACGTTTAGA	Construction of *pltA-Flag*
AG67-56-pltA-FLAG-F	CGCGGTACCATAGAGGAGGAAGAAATAATGAAAAAGTTAATA	Construction of *pltA-Strep*
AG67-87-pltA-Strep-R1	CGAACTGCGGGTGGCTCCAAGAACCTTTAGAAAGTATAAGTTCTATTACA	Construction of *pltA-Strep*
AG67-88-pltA-Strep-R2	CGCGAGCTCTTATTTTTCGAACTGCGGGTGGCTCCA	Construction of *pltA-Strep*
SH144-cyaA-pKD4-F	TATTGAGACTCTGAAACAGAGACTGGATGTGTAGGCTGGAGCTGCTTC	*cyaA* in-frame deletion
SH145-cyaA-pKD4-R	ATACTGCTGCAATAGCGGCGCGTCATGATCCATATGAATATCCTCCTTAG	*cyaA* in-frame deletion
SH126-dcyaA-F	TTTAAGAATTTACACGCAGCGAACGGTGCTAC	Sequence *cyaA* mutant
SH127-dcyaA-R	ACGCATCTGCCACCTCAAACCTCCTCCTGTAAA	Sequence *cyaA* mutant
RM79_cdtB_RTPCR_F	ACCTGGAATCTCGGAACCAC	qPCR detection of *cdtB*
RM80_cdtB_RTPCR_R	GGCGAGATGCGACAGTTGTC	qPCR detection of *cdtB*
RM83_pltB_RTPCR_F	GGTGTACCGCTACCGTTAGC	qPCR detection of *pltB*
RM84_pltB_RTPCR_R	TTAATGCCAGCGCTGAGTGG	qPCR detection of *pltB*
RM85_rpoB_RTPCR_F	TACGGGACGCATCCACGTAC	qPCR detection of *rpoB*

RM86_rpoB_RTPCR_R	GTGCGAACATGCAACGTCAG	qPCR detection of *rpoB*
RM298_artBqPCR_F	GAGTGAGGCTATTAGTGTCAATGCCAT	qPCR detection of *artB*
RM299_artBqPCR_R	TCCCTGGAAGAGAATGCACTTTTGA	qPCR detection of *artB*
